# Epitaxial Growth of Ga_2_O_3_: A Review

**DOI:** 10.3390/ma17174261

**Published:** 2024-08-28

**Authors:** Imteaz Rahaman, Hunter D. Ellis, Cheng Chang, Dinusha Herath Mudiyanselage, Mingfei Xu, Bingcheng Da, Houqiang Fu, Yuji Zhao, Kai Fu

**Affiliations:** 1 Electrical and Computer Engineering, The University of Utah, Salt Lake City, UT 84112, USA; u1351894@utah.edu (I.R.); u0973796@utah.edu (H.D.E.); 2 Department of Electrical and Computer Engineering, Rice University, Houston, TX 77005, USA; cc206@rice.edu (C.C.); mx9@rice.edu (M.X.); 3 School of Electrical, Computer and Energy Engineering, Arizona State University, Tempe, AZ 85281, USA; dinusha@asu.edu (D.H.M.); bingche1@asu.edu (B.D.); houqiang@asu.edu (H.F.)

**Keywords:** β-Ga_2_O_3_, epitaxial growth, MBE, MOCVD, HVPE, Mist CVD, PLD, LPCVD

## Abstract

Beta-phase gallium oxide (β-Ga_2_O_3_) is a cutting-edge ultrawide bandgap (UWBG) semiconductor, featuring a bandgap energy of around 4.8 eV and a highly critical electric field strength of about 8 MV/cm. These properties make it highly suitable for next-generation power electronics and deep ultraviolet optoelectronics. Key advantages of β-Ga_2_O_3_ include the availability of large-size single-crystal bulk native substrates produced from melt and the precise control of n-type doping during both bulk growth and thin-film epitaxy. A comprehensive understanding of the fundamental growth processes, control parameters, and underlying mechanisms is essential to enable scalable manufacturing of high-performance epitaxial structures. This review highlights recent advancements in the epitaxial growth of β-Ga_2_O_3_ through various techniques, including Molecular Beam Epitaxy (MBE), Metal-Organic Chemical Vapor Deposition (MOCVD), Hydride Vapor Phase Epitaxy (HVPE), Mist Chemical Vapor Deposition (Mist CVD), Pulsed Laser Deposition (PLD), and Low-Pressure Chemical Vapor Deposition (LPCVD). This review concentrates on the progress of Ga_2_O_3_ growth in achieving high growth rates, low defect densities, excellent crystalline quality, and high carrier mobilities through different approaches. It aims to advance the development of device-grade epitaxial Ga_2_O_3_ thin films and serves as a crucial resource for researchers and engineers focused on UWBG semiconductors and the future of power electronics.

## 1. Introduction

In power electronics, conventional Si-based transistors with a narrow bandgap of 1.12 eV are limited in high-voltage and high-temperature operations due to their intrinsic material properties [1]. Advances in semiconductor technology have paved the way for next-generation power devices using wide bandgap (WBG) semiconductors like SiC (3.2 eV) [2] and GaN (3.39 eV) [3], which offer superior electrical characteristics. GaN devices, for example, have significantly enhanced the bandwidth and reduced the power consumption compared to traditional technologies, enabling the development of monolithic circuits [1,4]. These improvements are mainly due to higher electrical breakdown fields of WBG semiconductors as their bandgaps increase. WBG semiconductors, such as GaN, have well-established growth processes and controllable n- and p-type doping, making them popular in electronic and optoelectronic applications [5,6,7]. At the same time, ultra-wide bandgap (UWBG) semiconductors with even wider bandgaps (>3.4 eV), including β-Ga_2_O_3_ (4.5–4.8 eV) [8], diamond (5.47 eV) [9], and AlN (6.2 eV) [10], have been extensively studied for applications in power electronics, extreme environment electronics, gas sensors, and UV detectors [11,12]. Among these materials, β-Ga_2_O_3_ is particularly notable for power electronics; for instance, the Baliga’s figure of merit (BFOM) of β-Ga_2_O_3_ is 136 GW·cm^−2^, which outperforms Si (by 3000×), SiC (by 10×), and GaN (by 4×) [13]. β-Ga_2_O_3_ is an attractive alternative for power devices because of its high breakdown field (8 MV/cm). Its growing prominence is also due to the rapid development of high-quality, large wafers up to 6 inches, making it suitable for mass production [14,15,16]. Ga_2_O_3_ has five different polymorphs, with β-Ga_2_O_3_ being the most thermally and chemically stable. Its wide bandgap makes it transparent to wavelengths below 250 nm, making it suitable for deep-UV optoelectronic applications. Polycrystalline β-Ga_2_O_3_ films with oxygen vacancies are widely researched as gas sensors for O_2_, CO, CH_4_, and H_2_. When these gases are adsorbed, they change the electrical conductivity of the film, making it useful for sensing applications [17,18,19]. The availability of commercially grown single-crystal substrates through melt methods has increased interest in using β-Ga_2_O_3_ for various technological applications [20,21]. These include transparent electrodes, thin-film transistors, gas sensors, solar-blind photodetectors, and UVC band LEDs [22]. However, β-Ga_2_O_3_-based devices suffer from poor thermal conductivity (~10–30 W/m·K), limiting their use in high-power, high-frequency applications due to localized heat generation, which significantly shortens device lifespan [23,24]. To address this issue, it is essential to incorporate heat sinks made from materials with high thermal conductivity, such as diamond (~2200 W/m·K) [25], near the active regions of these devices. Researchers have explored various methods to integrate β-Ga_2_O_3_ with diamond, including attaching exfoliated β-Ga_2_O_3_ onto single-crystal (SC) diamond, growing polycrystalline β-Ga_2_O_3_ on SC diamond, mechanically integrating the building blocks of β-Ga_2_O_3_ and diamond and bonding SC diamond wafers to β-Ga_2_O_3_ wafers at low temperatures [25,26,27,28,29]. Additionally, efforts have been made to grow diamonds on β-Ga_2_O_3_ using CVD, both with and without an interfacial layer [30,31,32]. Malakoutian et al. reported a thermal conductivity of 110 ± 33 W/m·K for the diamond film grown on β-Ga_2_O_3_ and a thermal boundary resistance of 30.2 ± 1.8 m^2^·K/GW at the diamond/β-Ga_2_O_3_ interface [30]. These findings suggest that further improvements in thermal management are needed, either by using an interfacial layer with higher thermal conductivity, eliminating the interfacial layer, or developing better techniques for creating diamond/β-Ga_2_O_3_ heterojunctions. For practical device applications, high-quality Ga_2_O_3_ is grown on native Ga_2_O_3_ substrates or foreign substrates such as c- or m-plane sapphires. Various polymorphs of epitaxially grown Ga_2_O_3_ have been observed, depending on the crystal-growth techniques, conditions, and substrates used. This indicates that the structural properties of epitaxial Ga_2_O_3_ are significantly influenced by the thermodynamics of the growth process and the conditions after growth. Epitaxial growth technologies for β-Ga_2_O_3_ have advanced significantly in the past decade, providing well-controlled and high-quality films for device designs. Various growth approaches, including molecular beam epitaxy (MBE), metal–organic chemical vapor deposition (MOCVD), halide vapor-phase epitaxy (HVPE), mist chemical vapor deposition (Mist-CVD), pulsed laser deposition (PLD), and low-pressure chemical vapor deposition (LPCVD) have been demonstrated, as indicated in Table 1 [33,34,35,36,37,38,39,40,41,42]. Notably, a record high room-temperature mobility of 194 cm^2^/V·s has been reported in an unintentionally doped β-Ga_2_O_3_ film grown on a Fe-doped (010) native substrate using MOCVD [43]. Although β-Ga_2_O_3_ should be electrically insulating without intentionally doping, experimentally grown Ga_2_O_3_ exhibits n-type conductivity with electron concentrations ranging from 10^14^ to 10^18^ cm^−3^, and its experimental mobility is still far below the theoretical value (μ = 300 cm^2^/V·s) [43,44,45]. Enhancing the growth rate of epitaxially grown β-Ga_2_O_3_ films without compromising crystal quality and electron transport remains a significant challenge. Issues such as poor crystal quality, background impurities, low career mobility, and low thermal conductivity, especially in thick epitaxial films, continue to pose major obstacles to achieving high-performance Ga_2_O_3_ power devices.

This review paper explores the prospects and current status of Ga_2_O_3_ epitaxial growth technologies, offering an up-to-date database of optimal growth conditions. We have explored various techniques, including MBE, MOCVD, HVPE, Mist-CVD, PLD, and LPCVD, with detailed discussions on each. Our goal is to provide readers with a comprehensive understanding of high-quality β-Ga_2_O_3_ epitaxial growth through different methods. This knowledge will contribute to advancing the development of high-quality Ga_2_O_3_ and its applications in power electronics.

Table 1 summarizes the physical properties and epitaxial growth methods for the five crystal phases of Ga_2_O_3_, while Table 2 compares the material properties of Ga_2_O_3_ with other semiconductors.

**Table 1:** Summary of physical properties and epitaxial growth methods for the five crystal phases of Ga_2_O_3_ [16,46,47,48,49,50,51,52,53,54,55].

Crystal Structure	Lattice Parameters	Space Group	Bandgap (eV)	Refractive Index (n)	Thermal Conductivity (W·cm^−1^·K^−1^)	Epitaxial Methods
α (Corundum)	a = b = 4.98–5.04, c = 13.43–13.62	R-3C	5.2–5.41	1.74–1.95	-	Mist CVD, MOCVD, HVPE
β (Monoclinic)	a = 12.12–12.34, b = 3.03 and 3.04, c = 5.80–5.87	C2/m	4.5–4.9	1.68–1.89	0.27 (010), 0.11 (100)	MBE, Mist CVD, MOCVD, PLD, LPCVD, sputtering, HVPE
γ (Cubic defective spinel)	a = 8.24–8.30	FD-3m	4.5–5.0	2.0–2.1	-	Mist CVD
Δ (Cubic bixbyite)	a = 9.4–10	Ia-3	4.8–5.0	1.8	-	Mist CVD
κ (Orthorhombic)	a = 5.0463, b = 8.7020, c = 9.2833	Pna21	4.6–4.9	-	-	MBE, Mist CVD, MOCVD, PLD, HVPE

**Table 2:** Material properties of Ga_2_O_3_ and other semiconductors [16,56,57].

Properties	Si	GaAs	4H-SiC	GaN	Ga_2_O_3_	Diamond
Bandgap, *E*_g_ (eV)	1.1	1.4	3.3	3.4	4.85	5.5
Breakdown field, *E*_c_ (MV·cm^−1^)	0.3	0.4	2.5	3.3	8	10
Electron mobility, *μ* (cm^2^·V^−1^·s^−1^)	1400	8000	1000	1250	300	2000
Relative dielectric constant, *ε*	11.8	12.9	9.7	9	10	5.5
Thermal conductivity, *λ* (W·cm^−1^·K^−1^)	1.5	0.5	4.9	2.3	0.1–0.3	20
BFOM, *εμE_c_*^3^ (GW·cm^−2^)	0.04	0.58	13.42	35.80	136.00	973.96

## 2. Molecular Beam Epitaxy (MBE)

### 2.1. Introduction

**Figure 1:**
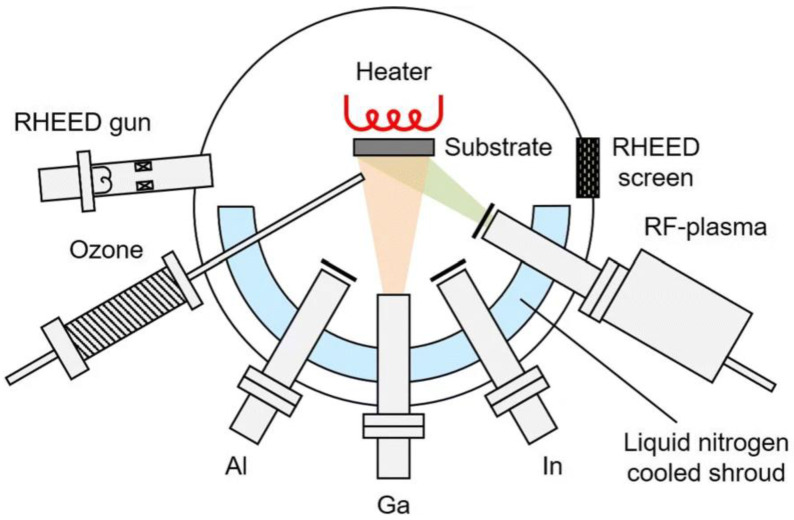
Schematic diagram of the MBE tool, showing the O_3_ inlet and O-plasma cell used to grow epitaxial β-Ga_2_O_3_ films (Reprinted with the permission of ref. [66]). Copyright 2022 under the terms of CC BY 4.0.).

Ever since its inception in the late 1960s, Molecular Beam Epitaxy (MBE) has built a strong reputation for producing materials with exceptional structural, optical, and electrical characteristics. It has become one of the most extensively researched and potentially commercialized techniques for depositing β-Ga_2_O_3_ thin films due to its remarkable ability to create extremely pure and defect-free epilayers. This is made possible by maintaining an ultra-high vacuum environment during growth and using exceptionally pure metal and oxygen sources. Figure 1 shows a diagram of a typical MBE machine used for growing Ga_2_O_3_. In this process, an effusion cell heats a Ga metal source to produce a molecular beam flux that travels directly toward a substrate. Ga_2_O_3_ grows on the substrate when the Ga atoms are oxidized using oxidants like ozone (O_3_) or oxygen (O) radicals. During Ga_2_O_3_ MBE growth, two key reactions occur: first, Ga atoms are oxidized on the surface, forming volatile gallium suboxides (Ga_2_O). In the second stage, Ga_2_O either evaporates from the surface or is further oxidized into Ga_2_O_3_, which becomes part of the growing layer. This growth process is highly influenced by factors such as the O/Ga flux ratio, growth temperature, and substrate orientation, as the balance between Ga_2_O desorption and oxidization controls the growth rate. Various types of devices, such as photodetectors, Schottky diodes, and MOSFETs, have been reported using MBE-grown Ga_2_O_3_ [58,59,60,61,62]. While the study of the materials system is still in its early stage, plasma-assisted molecular beam epitaxy (PAMBE) has successfully produced β-Ga_2_O_3_ with various doping and heterostructure schemes [63]. On the other hand, ozone-enhanced MBE (ozone MBE) allows for examining the relationship between surface orientation and film quality, as well as the application of various doping choices [64,65]. In this session, we will discuss the current status and challenges of MBE-grown Ga_2_O_3_.

### 2.2. Effects of Growth Conditions on Film Properties

In a previous comprehensive study, people aim to elucidate the intricate relationship between growth conditions and key film properties in β-Ga_2_O_3_ thin films. In Section 2.2, we tend to review the effects of growth conditions on these film properties of β-Ga_2_O_3_ thin film: morphology, growth rate, and thermal conductivity. This investigation is vital for advancing our understanding of thin-film deposition processes and optimizing the performance of β-Ga_2_O_3_ films in diverse applications ranging from optoelectronics to thermal management.

#### 2.2.1. Morphology

Morphology plays an important role in device fabrication; for example, there are smoother interfaces in (010) β-Ga_2_O_3_/(Al_x_Ga_1−x_)_2_O_3_ heterostructures, whose roughness is ruled by that of the β-Ga_2_O_3_ layer, which can enable higher-mobility 2-dimensional electron gases by reducing interface roughness scattering [67]. Morphology was affected by many growth parameters, and lots of work has been done to explore by changing one or two parameters.

1. Substrate

Epitaxial growth onto Si (001) wafers would pave the way for numerous possibilities in large-scale integration. Tobias et al. used SrTiO_3_ (001) as a buffer layer on Si to prevent Si oxidation and reaction between Ga_2_O_3_ and Si during growth. The buffer layer can also act as a template to guide oxide-on-oxide epitaxial growth [68]. If epitaxial growth is directly performed on Ga_2_O_3_ substrates, the direction of substrates may also affect the surface morphology a lot. In general, the (010) plane is associated with the highest growth rates, but for achieving an atomically flat surface, the (110) orientation is preferable due to its energetically stable surface and step-flow growth. Takeki et al. conducted a study on the surface morphology of β-Ga_2_O_3_ growth on (110) substrates using PAMBE while using (010) substrates as references under identical growth conditions [69]. Their findings revealed that despite the presence of (110) facets in the growth of (010) β-Ga_2_O_3_, the (110) plane did not exhibit a well-defined step–terrace structure. Additionally, they observed that surface morphologies were influenced by the Ga flux, which will be elaborated upon later.

2. Growth temperature

**Figure 2:**
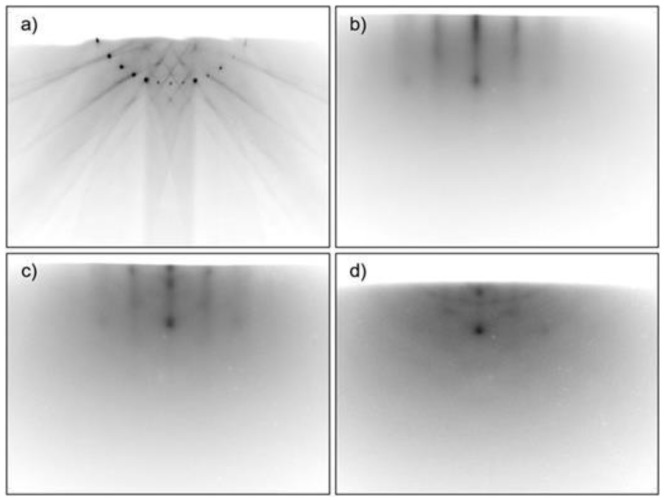
RHEED patterns at different stages of growth: (**a**) Si (001) 2 × 1 surface after SiO_2_ desorption with Sr along the Si [110] azimuth (2 in. holder). (**b**) Postgrowth surface of 5 nm e-beam-evaporated γ-Al_2_O_3_ (111) grown at 840 °C along the Si [72] azimuth with 12-fold rotational symmetry (2 in. holder). (**c**) The post-growth surface of 21 nm Ga_2_O_3_ grown at 670 °C along the Si [72] azimuth (2 in. holder). (**d**) The post-growth surface of additional 45 nm Ga_2_O_3_ grown at 630 °C along the Si [72] azimuth (10 × 10 mm^2^ holder) [71]. Copyright (2021) AIP publishing.

Optimal thermal conditions play a crucial role in attaining the preferred crystalline quality, structural integrity, and material properties throughout the growth process. Trong et al. found the surface morphology changed at growth temperatures of 550 to 800 °C [70]. The RMS increased and reached the maximum value at 650 °C and decreased with the increasing growth temperature. The surface morphology and roughness depend on the correlation of lateral and vertical growth rates. At lower temperatures, the vertical growth rate dominates, which leads to the granular surface. As the temperature increases, the lateral growth rate increases and gradually dominates, which makes the larger grain size with smooth two-dimensional features. Tobias et al. grew β-Ga_2_O_3_ on Si with γ-Al_2_O_3_ (111) as the buffer layer and compared high-energy electron diffraction (RHEED) at 670 °C and 630 °C [71]. As Figure 2 shows, the sample at 630 °C displays a less streaky pattern with strong modulation along the streaks, which is related to a decrease in crystalline quality at the lower growth temperature.

3. Ga flux

**Figure 3:**
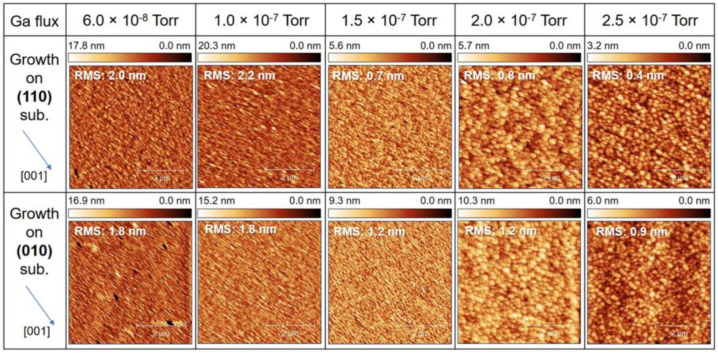
AFM images of β-Ga_2_O_3_ epitaxial films grown on (110) and (010) substrates at different Ga fluxes at 700 °C are measured for a scan area of 5 × 5 μm^2^. Elongated features along the [001] direction can be observed on both β-Ga_2_O_3_ epitaxial films grown on (110) and (010) substrates [69]. Copyright (2020) AIP publishing.

Ga flux can be controlled by providing various Ga-effusion cell temperatures. In their study, Trong et al. observed that the surface morphology and roughness of Ga_2_O_3_ films remained relatively unchanged despite variations in the Ga/O ratio caused by adjustments in the Ga cell temperature [70]. They noted that the RMS roughness exhibited an increase in response to the Ga flux, although the change was not considered to be drastic. However, in Takeki et al.’s work, they conducted a more detailed analysis of Ga flux and surface morphology [69]. As Figure 3 shows, there are pits on the surface at Ga fluxes from 3.0 × 10^−8^ to 1.5 × 10^−7^ Torr. But at a higher Ga flux, smoother surfaces without pits are achieved, and the crystal size of β-Ga_2_O_3_ becomes larger due to the high and stable growth rate.

4. Other factors

**Figure 4:**
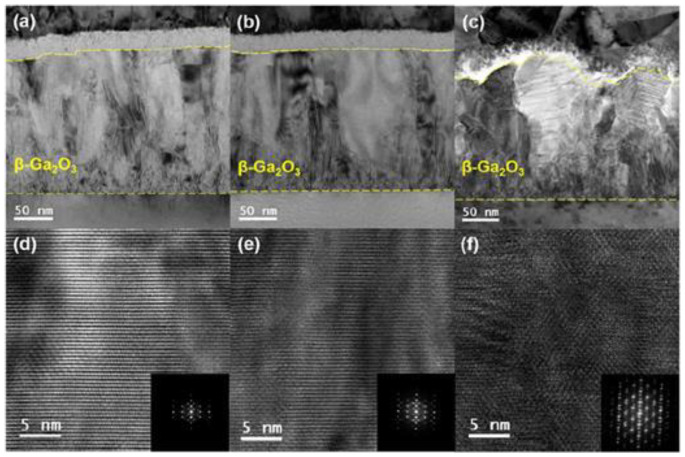
TEM images of β-Ga_2_O_3_ films with (**a**) UID; (**b**) Si-dopped at 4.42 × 10^19^ cm^−3^; (**c**) Si-dopped at 6.11 × 10^20^ cm^−3^. The corresponding magnified images and FFTs (**d**–**f**) [73]. Copyright (2024) AIP publishing.

Si has been recognized as the best choice for n-type doping for β-Ga_2_O_3_ application, as it is the only true shallow donor and has excellent electrical properties. Jiali et al. found that the quality of Si dopant impacts the crystallinity of the films [73]. From the TEM analysis in Figure 4, we can find that film with Si at a concentration of 4.42 × 10^19^ cm^−3^ exhibits a pristine single-crystal morphology. However, as the concentration increases, a wider range of crystal orientations and a more uneven surface texture appear. The concentration will lead to a transition from single crystal to polycrystalline morphology.

Morphology can also be affected by other factors, like precursors. Chengyi et al. explored the effect of oxygen precursors on β-Ga_2_O_3_; they elucidated the impact of ozone and oxygen plasma precursors on the growth mechanism [74]. The use of different precursors results in distinct growth modes, known as Volmer–Weber and Stranski–Krastanow. When only ozone precursors are used, samples have a limited number of nucleation sites, resulting in a small grain size. Over time, the number of nucleation sites increases, contributing to heightened surface roughness. Conversely, samples using oxygen precursors display a high density of nucleation sites in the early stages. As these sites combine over time, they form large, oriented grains, leading to a gradual reduction in roughness. Rie et al.’s work also further demonstrated that ozone has a different growth mechanism with PAMBE [75].

#### 2.2.2. Growth Rate

**Figure 5:**
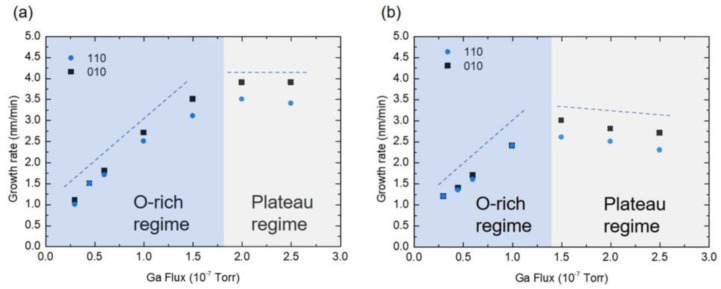
(**a**) Growth rates of β-Ga_2_O_3_ epitaxial films grown on (110) and (010) substrates at 600 °C and (**b**) 700 °C [69]. Copyright (2020) AIP publishing.

As mentioned above, the Ga flux controls the morphology of the final film by affecting the growth rate. In Takeki et al.’s work, they found the dependence of the growth rate on Ga flux demonstrated two clear growth regimes [69]. The O-rich regime is where the growth rates increase linearly with the Ga flux. At higher Ga fluxes, growth transitions to the plateau regime where the growth rate remains constant, as Figure 5 shows.

The maximum growth rate achieved in the plateau regime is also dependent on growth temperature. Patrick et al. provided a reaction-rate model describing the growth of Ga_2_O_3_ [76]. The growth rate can be expressed as Equation (1) [76]:

(1) $$\frac{{\mathrm{dn}}_{{\mathrm{Ga}}_{2}O_{3}}}{\mathrm{dt}}={\;\kappa }_{{\mathrm{Ga}}_{2}O}n_{{\mathrm{Ga}}_{2}O}n_{O}^{2}$$

The Ga_2_O_3_, Ga_2_O, and O adsorbate densities are denoted as nGa_2_O_3_, $n_{{\mathrm{Ga}}_{2}O}$_O_, and $n_{O}$, respectively. The flux of available O adsorbates, for Ga_2_O to Ga_2_O_3_ oxidation at a particular T_G_, is determined by its sticking coefficient σ on the Ga_2_O_3_ growth surface and is described by Equation (2) [76]:

(2) $$\sigma (T_{G})=(1+\sigma_{0}\;\exp\;(-\frac{\Delta \sigma }{k_{B}T_{G}}){)}^{-1}$$

Equation (2) reflects the decreasing probability of O species to adsorb as T_G_ is increased, which leads to a decrease in growth rate. Suboxide formation is a limiting feature of the growth rate due to the preferred formation of volatile Ga_2_O suboxide over Ga_2_O_3_ at higher growth temperatures. In Akhil et al.’s work, they reported on metal oxide catalyzed epitaxy (MOCATAXY), using Indium as a catalyst, to improve growth rates in PAMBE-grown β-Ga_2_O_3_ thin films [77]. The enhanced growth rate could be attributed to an In or indium oxide catalyst layer forming at the surface of the substrate during growth that could react with some molecular O_2_ and atomic O in the growth chamber, which can reduce suboxide desorption.

#### 2.2.3. Thermal Conductivity

**Figure 6:**
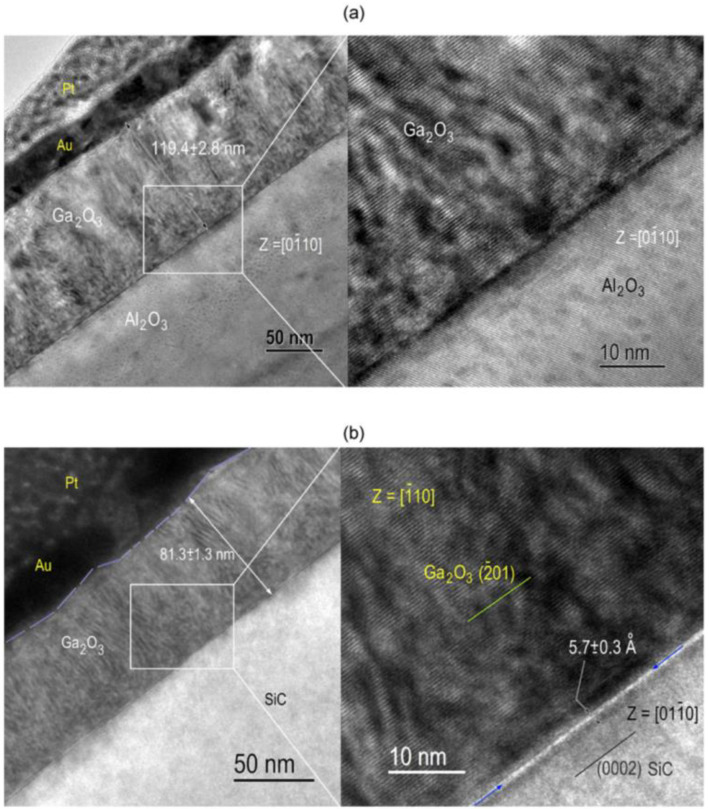
Cross-sectional phase contrast TEM micrographs of (**a**) β-Ga_2_O_3_ on c-sapphire showing the average film thickness of ~119.4 ± 2.8 nm, and to the right-hand side is a high-resolution lattice image of the showing perfectly sharp interface and (**b**) β-Ga_2_O_3_ on SiC with an average thickness of ~81.3 ± 1.3 nm, and a thin crystalline interfacial SiOx layer [80]. Copyright (2020) AIP publishing.

The maximum reported bulk thermal conductivity of Ga_2_O_3_ at 300 K is around 26 W/(m·K) in the (010) direction, which will hinder the full potential of Ga_2_O_3_-based devices [78]. Some research has been done to overcome this limitation for using β-Ga_2_O_3_ in high-frequency and power-switching applications. The growth of Ga_2_O_3_ thin films on high thermal conductivity foreign substrates can provide a pathway for developing power devices. Neeraj et al. first report the growth of β-Ga_2_O_3_ films grown by MBE on 4H-SiC [79]. The thermal conductivity of an 81 nm-thick Ga_2_O_3_ layer on 4H-SiC has been measured at 3.1 ± 0.5 W/m·K, which is lower than that of the same layer grown on sapphire. However, thermal conductivity values of MBE-grown Ga_2_O_3_ films show a slight increase, with reported values of about 2.9 W/m·K, compared to similar thickness films grown by pulsed laser deposition on c-sapphire. They confirmed that the thermal conductivity depends on the deposition method since the photons could scatter on deposition-induced crystal defects. It can be seen in Figure 6 that the β-Ga_2_O_3_/SiC interface has a metastable SiOx layer that is polycrystalline in structure, which was not seen on the β-Ga_2_O_3_/sapphire interface.

In Diego et al.’s work, they grew β-Ga_2_O_3_ with lower thermal conductivity by MBE and compared experimental values against the numerical predictions to decipher the effect of boundary scattering and defects [80]. In their analysis, they found that the sample’s thermal conductivity was three times smaller than the numerically calculated conductivity for films of similar thickness but without defects. They examined the percentage of vacancies of gallium and oxygen atoms, linear defects in thin films, and the lateral grain boundaries to explain the experimental results. The presence of grain-boundary scattering contributed to a 32% reduction in thermal conductivity, 1% of Ga vacancies contributed to a 28% reduction, and the existence of 106 cm^−1^ linear defects led to a 30% reduction in thermal conductivity.

### 2.3. Doping

Precise control of its electrical properties is essential to harness the potential of β-Ga_2_O_3_ in power electronic applications fully. Due to the absence of p-type doping for β-Ga_2_O_3_, the primary focus lies in utilizing Group IV elements to regulate n-type doping. Among these elements, silicon stands out as the most effective donor, being the only truly shallow donor. Its ability to modulate the mobile carrier density enables a remarkable change of over five orders of magnitude in the electrical resistivity, coupled with a mobility of 390 cm^2^/V·s [81]. However, it has been noticed that Si doping in β-Ga_2_O_3_ is relatively uncontrolled, only having a low concentration side of the doping window [65]. Another issue is the donor density did not change with dopant effusion cell temperature [82]. To realize or further improve the aforementioned devices, it is of essential importance to have the ability to grow high-quality Ga_2_O_3_ thin films with controlled doping concentrations over a wide range. Zhuoqun et al. proposed Si doping using diluted disilane as a gas source in a hybrid MBE system [83]. They found that Si incorporation did not depend on the growth temperature, ranging from 550 to 700 °C, but it would increase to 525 °C. Based on that, a wide range of Si doping concentrations (1 × 10^16^–2 × 10^19^ cm^−3^) was obtained. To overcome MBE’s shortcoming in the growth rate, Kathy et al. utilized pre-oxidized gallium in the form of a molecular beam, facilitating Ga_2_O supply to the growth surface. This method bypassed the rate-limiting first step of the two-step reaction by suboxide molecular-beam epitaxy [84]. SiO suboxide produced by Silicon-containing oxide sources was used to dope the film. The doping was controlled in the 5 × 10^16^–10^19^ cm^−3^ range, and the growth rate could reach 1 μm/h.

**Figure 7:**
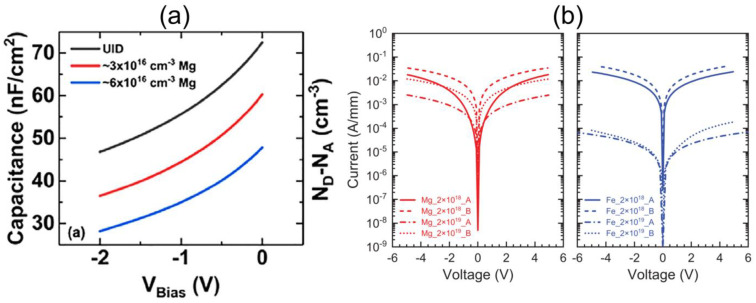
(**a**): Comparison of unintentionally doped and Mg co-doped samples in terms of capacitance-voltage characteristics at 100 KHz. (**b**) Leakage current characteristics of Mg- and Fe-implanted test structures [85,86]. Copyright (2020) AIP publishing.

Si doping can improve electrical properties, but the highly Si-doped region around the interface typically becomes a current conduction path, causing buffer leakage. Ashok et al. developed in situ Mg doping techniques in plasma-assisted molecular beam epitaxy (PAMBE) of β-Ga_2_O_3_ to compensate Si dopants at the substrate epilayer growth interface and eliminate parasitic leakage paths [85]. From the C-V characteristics in Figure 7a, it can be confirmed that the introduction of Mg dopants can provide an effective way to compensate donors in β-Ga_2_O_3_ layers grown by PAMBE. However, Sandeep et al. performed Mg- or Fe-ion implantation doping into a Ga_2_O_3_ before MBE to overcome the leakage and obtain an opposite conclusion [86]. From Figure 7b, we can find that Mg-implantation doping showed a minimal reduction in interface leakage, irrespective of its concentration. However, the Fe doping with a high density of 2 × 10^19^ cm^−3^ provided a significant decrease in the leakage and decent FET characteristics. In previous works, Mg doping has been demonstrated in MOCVD or by ion implantation, but significant diffusion was observed after annealing at 800 °C to recover ion implantation damage [87]. In Akhil’s work, they investigated the doping and diffusion of Mg in β-Ga_2_O_3_ films grown by PAMBE, and Mg concentrations from 2 × 10^16^ to 8 × 10^20^ cm^−3^ with sharp doping profiles were realized. They found that annealing at 925 to 1050 °C resulted in significant diffusion, so a wide range of Mg doping concentrations can be achieved with sharp doping profiles at the lower temperature. In the study, Akhil et al. investigated the Sn doping of β-Ga_2_O_3_ films using MOCATAXY. Their work resulted in the achievement of a wide range of Sn doping concentrations, as well as sharp, controllable profiles [88]. They obtained a relatively high room temperature mobility of 136 cm^2^/V·s.

### 2.4. Summary and Future Prospects

**Figure 8:**
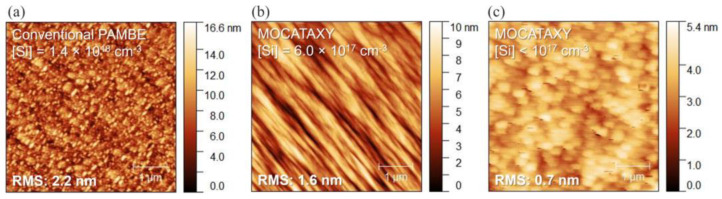
(**a**) AFM images of Si-doped β-Ga_2_O_3_ (010) films grown by conventional MBE and (**b**,**c**) MOCATAXY [90]. Copyright (2023) AIP publishing.

The molecular beam epitaxy (MBE) method is widely known for its ability to produce high-quality, defect-free thin films of β-Ga_2_O_3_ and allows for precise control over n-type doping. Furthermore, the MBE technique enables real-time observation of surface structure and morphology with atomic layer precision using Reflective High-Energy Electron Diffraction (RHEED), making it possible to effectively study the mechanisms of crystal growth and dopant diffusion. As a result, it has become one of the most extensively researched and potentially commercialized techniques for β-Ga_2_O_3_ thin-film deposition. Nevertheless, it still faces challenges such as low growth rates and small film sizes. In addition to the aforementioned work, new growth methods based on MBE have also emerged in recent years to address the limitations of the growth process. Mazzolini et al. further demonstrated and summarized the epitaxial growth rate for different crystal planes of β-Ga_2_O_3_ by using indium-mediated metal-exchange catalyzed MBE (MEXCATMBE) [89]. They found that the growth rate of the In-catalyzed layers increases with the surface-free energy of the β-Ga_2_O_3_ substrate. Takeki et al. performed metal-oxide catalyzed epitaxy (MOCATAXY) to improve the crystal quality [90]. In Figure 8, it is evident that the films produced with MOCATAXY exhibited smaller RMS roughness compared to the films grown using conventional MBE. Due to the deepening of further research, the utilization of β-Ga_2_O_3_ is anticipated to have a more extensive future.

## 3. Metalorganic Chemical Vapor Deposition (MOCVD)

### 3.1. Introduction

Metal-organic chemical Vapor Deposition (MOCVD) is the preferred standard method for manufacturing epitaxial layers, primarily used in creating III–V, III–VI, and II–VI-based optoelectronic and power devices. Recent advancements in MOCVD have achieved significant milestones in the growth of β-Ga_2_O_3_ films. Notably, the highest recorded growth rate stands at 10 µm/h, alongside the lowest observed point-defect concentration [91]. Remarkably, room temperature mobility has been reported to range between 150 and 194 cm^2^/V·s with a background impurity concentration of 10^14^ to 10^16^ cm^−3^ [92,93,94,95,96], and the lowest free-carrier concentration noted was 2.4 × 10^14^ cm^−3^ in nitrogen-doped β-Ga_2_O_3_ films [96]. Elements like silicon (Si), tin (Sn), and germanium (Ge) have been effective as shallow donors [92,93,97]. Experiments using a vertical far-injection reaction chamber showed that high-quality undoped intrinsic (UID) Ga_2_O_3_ homoepitaxy achieved room-temperature mobility of 176 cm^2^/V·s with a background concentration of 7 × 10^15^ cm^−3^ [94]. Additionally, lightly doped (010) Ga_2_O_3_ demonstrated peak mobility exceeding 10^4^ cm^2^/V·s at a low temperature of 45 K as measured by the Hall effect, highlighting the ultrahigh purity of the MOCVD-grown Ga_2_O_3_ [98]. The growth rate, crystal quality, background impurities, and extended defects depend significantly on factors such as the native substrate orientation, growth precursors, growth parameters, and the reactor environment. Cooke et al. identified a novel structural defect in MOVPE-grown homoepitaxial β-Ga_2_O_3_ films, referred to as “sympetalous defects”, which are closely associated with pre-existing nanotube defects in the substrate [99]. These defects have a substantial impact on the quality and performance of epitaxial films. To mitigate their effects, Cooke et al. emphasize the importance of understanding and controlling these defects, as they can significantly influence local film properties and lead to issues such as leakage currents and reduced device reliability. Chou et al. commonly observed parasitic particles in films thicker than 1.5 μm, which are primarily attributed to gas-phase reactions in the chamber [100]. These particles can induce structural defects, such as twin lamellae, which in turn degrade carrier mobility and other electrical characteristics of the grown films. To mitigate this issue, strategies such as increasing the total gas flow and reducing the showerhead distance have been shown to significantly decrease the density of parasitic particles [101]. These adjustments help reduce the residence time of precursors, thereby limiting gas-phase reactions that lead to particle formation. In addition, optimizing the gas-flow distribution in the chamber has been shown to further suppress particle nucleation, resulting in higher-quality films with improved mobility. Additionally, Chou et al. studied the influence of the O_2_/Ga ratio on morphological stability in MOVPE-grown β-Ga_2_O_3_ films, providing valuable insights into morphological instability when growing thick layers along the (100) orientation of β-Ga_2_O_3_ [102]. They found that a high O_2_/Ga ratio can lead to significant morphological instabilities, including step meandering and bunching, as the film thickness increases. These instabilities are particularly problematic when aiming to grow super-thick layers, as they can degrade the surface quality and overall electrical properties of the film. Conversely, reducing the O_2_/Ga ratio has been shown to effectively suppress these instabilities, allowing for the growth of thicker, more morphologically stable films.

Understanding these fundamental material properties and systematically mapping the growth processes are crucial for achieving the desired outcomes. Notably, the highest room temperature mobility, crystal perfection, and the lowest background impurity concentrations vary with different growth rates and tend to degrade as the growth rate increases. One of the primary challenges in this field is enhancing the growth rate without compromising material quality. Optimizing nucleation processes and managing the adsorption/desorption of adatoms, along with their diffusion lengths on native substrate surfaces, are critical for improving crystal quality. Additionally, the purity of materials is also influenced by the choice of growth precursors, the cleanliness of the reactor, and the specific growth conditions.

In the realm of MOCVD for growing β-Ga_2_O_3_ films, a variety of precursors have been utilized, including Triethylgallium (TEGa), Trimethylgallium (TMGa), and Ga(DPM)_3_ (where DPM stands for dipivaloylmethanate), as well as high-purity gases like O_2_, H_2_O vapor, and N_2_O [92,93,94,103]. A novel approach in MOCVD systems for Ga_2_O_3_ involves a close-coupled showerhead design that enhances growth rates. This design minimizes the distance between the showerhead and the substrate holders, effectively reducing the gas-phase reactions between metal-organic sources and oxygen, which in turn facilitates a rapid growth rate of 10 µm/h on sapphire substrates [91]. Various precursors are used in MOCVD for growing β-Ga_2_O_3_ films, including Triethylgallium (TEGa), Trimethylgallium (TMGa), Ga (DPM)_3_ (where DPM stands for dipivaloylmethanate), and high-purity gases like O_2_, H_2_O vapor, and N_2_O. A novel approach in MOCVD systems for Ga_2_O_3_ involves a close-coupled showerhead design that enhances growth rates. This design minimizes the distance between the showerhead and the substrate holders, reducing gas-phase reactions between metal-organic sources and oxygen, allowing for a rapid growth rate of 10 µm/h on sapphire substrates. TMGa allows for faster growth rates, but using TEGa for Ga_2_O_3_ films is challenging, especially as the film gets thicker. This makes it hard to develop the thicker layers needed for vertical-power devices. Typically, the growth rate for high-quality Ga_2_O_3_ films using TEGa is about 1 µm/h [43,92]. Furthermore, MOCVD growth has successfully produced β-Ga_2_O_3_ with high crystalline quality and effective n-type doping. A significant advancement within this technology is the high-quality epitaxy of β-(Al_x_Ga_1−x_)_2_O_3_, with aluminum incorporation rates of up to 40% on native (010) substrates. These developments underscore the considerable potential of the MOCVD growth method in creating high-quality β-Ga_2_O_3_-based thin films and heterostructures, which are crucial for high-performance device applications.

### 3.2. Chemical Kinetics of the MOCVD

**Figure 9:**
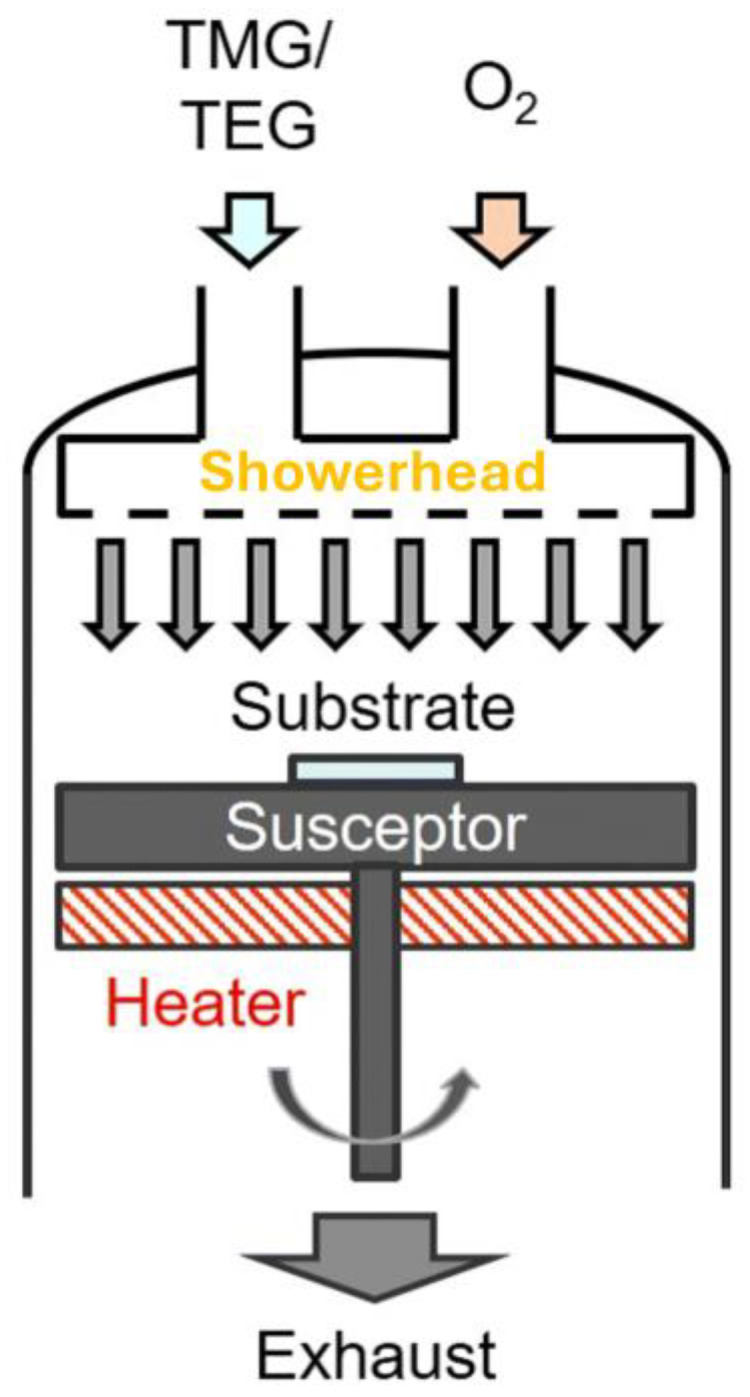
Schematic diagram of a typical MOCVD reactor for the growth of β-Ga_2_O_3_ (reproduced with permission from Ref. [66]. Copyright 2022 under the terms of CC BY 4.0.).

Figure 9 illustrates a typical MOCVD reactor for growing β-Ga_2_O_3_, highlighting its essential components. These components include the gas- or precursor-delivery system, source-injection control, a showerhead, the main reactor chamber equipped with an RF (radio frequency) heating coil, and pressure-control units connected to a high-capacity pump. Key features of the MOCVD reactor design include the management of volatile precursors and ensuring a uniform and consistent gas flow to the substrates. Additionally, precise control over reactor pressure is crucial, as is the effective heating and cooling of reactor components to establish the desired temperature gradient. The system also incorporates fast-switching valves that allow for the precise alternation between different sources, facilitating the accurate construction of multilayer structures. These specialized features ensure that the MOCVD system can meet the exacting requirements needed for high-quality epitaxial growth. For effective control of multilayer growth in MOCVD, precise and timely gas switching is crucial. This ensures that changes in the source material are immediately reflected on the growth surface. The precursor-delivery system in MOCVD typically consists of an array of high-purity metal-organic (MO) precursors, each contained in individual bubblers. Each bubbler is meticulously configured with its own temperature settings, carrier gas flow, and pressure controls to manage the delivery of the precursors.

The process of source injection is governed by a critical component known as the injection block, which controls the flow of sources into the system. The showerhead functions as a gas distributor, taking source gases from pipelines and transforming them into a stable, homogeneous flow. This ensures uniform distribution of the source gases across the growth surface. The design of the reactor chamber, including its geometry and the typical chamber pressure, plays a significant role in this process. With the right design parameters, MOCVD can produce high-quality, uniform thin films across large wafers. In some advanced designs, the substrates are placed on a fast-rotating susceptor, which further enhances the uniformity of the film growth by ensuring even distribution of heat and reactant gases over the substrate surface. These design features are essential for achieving the precision required in sophisticated semiconductor and optical device manufacturing. The general process of reaction and deposition in MOCVD involves several critical steps [104,105,106]:

Transport of Precursors: The precursors are transported to the reactor in vapor form.

Gas-phase Pre-reactions: This includes the pyrolysis of the precursors and the formation of adducts, which are complexes formed between different chemical species.

Mass Transport to Substrate: The chemically active species are transported to the substrate surface.

Adsorption: The reactants adsorb onto the substrate surface, initiating the chemical processes necessary for film growth.

Surface Diffusion and Reaction: Adatoms (adsorbed atoms) diffuse across the substrate surface to growth sites, where nucleation (the initial phase of growth) and surface chemical reactions occur, leading to the deposition of thin films.

Desorption and Mass Transport: Precursor fragments and by-products desorb from the substrate surface and are transported away.

Understanding these steps is crucial for optimizing the MOCVD process, particularly in enhancing the quality and uniformity of the resulting thin films. The growth characteristics of MOCVD, such as reaction rates, deposition rates, uniformity, and impurity incorporation, are governed by various physiochemical kinetics. These kinetics are highly dependent on several key growth parameters, including temperature, pressure, gas flow, and chemical concentrations. A critical factor influencing the deposition rate is the growth temperature, which can be categorized into three distinct regions:

Lower Growth Temperatures: Here, the deposition rate is predominantly governed by chemical-reaction kinetics. At this stage, the rate at which the surface reacts with chemical compounds rises exponentially with increasing substrate temperatures, in accordance with the Arrhenius law. This reflects how temperature influences the speed of chemical reactions at the substrate surface.

Intermediate Growth Temperatures: As the temperature increases further, the deposition rate becomes primarily constrained by mass transport. This involves the number of chemical species that can reach the growth surface or the diffusion of these species across the surface.

High Growth Temperature: At very high temperatures, the growth rate may start to decline. This decrease is due to the depletion of precursors in the gas phase, which happens because the boundary layer near the substrates heats up sufficiently to cause significant pre-reaction of the precursors. Additionally, processes like surface desorption and decomposition of the materials are accelerated under high temperatures, further reducing the growth rate.

In the case of TEGa, the decomposition primarily occurs through a three-step β-hydride elimination reaction. This complex reaction pathway leads to the formation of gallium hydride and ethylene as by-products. These processes are outlined in Equations (3)–(6) and detail the breakdown of TEGa and other related reactions involving TMGa (the Trimethylgallium), showcasing the intricate interactions at play during the deposition of β-Ga_2_O_3_ [55,107].

Ga(C_2_H_5_)_3_ → HGa(C_2_H_5_)_2_ + C_2_H_4_, (3)

Ga(C_2_H_5_)_2_ → H_2_Ga(C_2_H_5_) + C_2_H_4_, (4)

H_2_Ga(C_2_H_5_) → GaH_3_ + C_2_H_4_. (5)

The reaction between gallium hydride and oxygen (O_2_) to form gallium oxide (Ga_2_O_3_) typically proceeds as follows:

2GaH_3_ +3O_2_ → Ga_2_O_3_ +3H_2_O. (6)

Trimethylgallium (TMGa) undergoes a series of reactions where it progressively loses methyl groups, leading to the formation of simpler gallium methyl compounds. This process can be broken down into a two-step unimolecular reaction:

Ga(CH_3_)_3_ → Ga(CH_3_)_2_ + CH_3_, (7)

Ga(CH_3_)_2_ → Ga(CH_3_) + CH_3_. (8)

Although there is a scarcity of detailed studies on the subsequent reactions of these TMGa decomposition products with oxygen (O_2_) to form gallium oxide (Ga_2_O_3_), it is possible to hypothesize the following reaction pathway [55]:

2Ga (CH_3_) +3O_2_ → Ga_2_O_3_ +3 H_2_O +2CO_2_. (9)

During the growth process of β-Ga_2_O_3_ in MOCVD, by-products like hydrogen and hydrocarbons are turned into carbon monoxide (CO), carbon dioxide (CO_2_), and water (H_2_O) through combustion. These reactions indicate that the likelihood of carbon incorporation into MOCVD β-Ga_2_O_3_ is lower compared to when producing III–V compounds and III nitrides. Kinetic analyses suggest that increasing the growth temperature and maintaining a higher VI/III ratio can further reduce the chances of carbon incorporation into the film. It is also observed that the reactions between the precursors and oxygen (O_2_) are highly active. This leads to the inference that the mass transport of gallium (Ga) species to the growth surface acts as one of the primary limiting factors in the deposition of β-Ga_2_O_3_. Therefore, suppressing these gas-phase reactions can potentially increase the deposition rate of β-Ga_2_O_3_. The decomposition of TMGa through a two-step reaction, compared to the three-step pyrolysis process of TEGa, implies a shorter reaction pathway. This shorter pathway contributes to higher growth rates of Ga_2_O_3_ films when using TMGa as the gallium precursor. Specifically, growth rates for β-Ga_2_O_3_ range from 0.2 to 1.0 μm/h when TEGa is used, whereas using TMGa can achieve faster growth rates up to 10 μm/h. Despite the faster rates with TMGa, the transport characteristics of the films are comparable to those grown using TEGa, highlighting an efficient balance between growth speed and film quality [108,109].

### 3.3. Effects of Growth Conditions on Film Properties

#### 3.3.1. Effects of Growth Temperature

**Figure 10:**
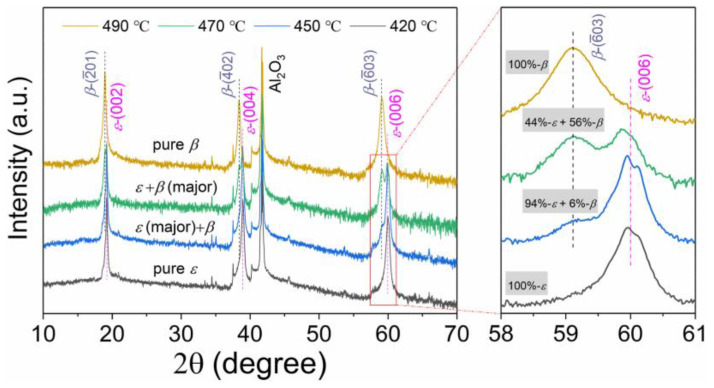
Displays the X-ray diffraction (XRD) patterns of Ga_2_O_3_ films grown at temperatures between 420 °C and 490 °C using MOCVD, revealing diverse phase compositions (reprinted with the permission of ref. [115]). Copyright 2022 under the terms of Elsevier publishing.

The growth temperature plays a vital role in the diffusion of adatoms (adsorbed atoms) on the growth surface, significantly influencing the surface morphology of β-Ga_2_O_3_ [93]. At lower growth temperatures (around 800 °C), the surface of the films tends to be rougher with visible macroscopic defects. In contrast, a temperature of 880 °C typically results in a uniform and smooth surface [92]. Further research into the homoepitaxial growth of β-Ga_2_O_3_ films suggests that even lower temperatures (below 700 °C) can yield films with atomically flat surfaces and satisfactory transport characteristics, demonstrating a broad range of suitable growth temperatures for MOCVD [96]. Additionally, implementing a low temperature (600 °C) buffer layer has been shown to improve the Hall electron mobility in both unintentionally doped and Si-doped β-Ga_2_O_3_ films [110]. Researchers have found that the temperature during the growth process significantly affects film quality. At excessively low temperatures, the movement of deposited particles across the surface is restricted, which prevents the formation of smooth, uniform films. Conversely, at very high temperatures, more particles form in the vapor above the surface rather than on it, which also degrades film quality [111,112,113]. Therefore, both very low and very high temperatures are detrimental to producing high-quality films. Li et al. achieved the FWHM of 21.6 arcsec for β-Ga_2_O_3_ homoepitaxy at the growth temperature of 750 °C with a surface RMS roughness of 0.68 nm using TMGa as a gallium precursor [114]. Yue et al. demonstrated that controlling the low temperature can induce a phase transition in Ga_2_O_3_ films grown by MOCVD. Specifically, adjusting the growth temperature from 420 to 490 °C results in a complete transformation from the epsilon (ε) phase to the beta (β) phase, as indicated in Figure 10 [115].

**Figure 11:**
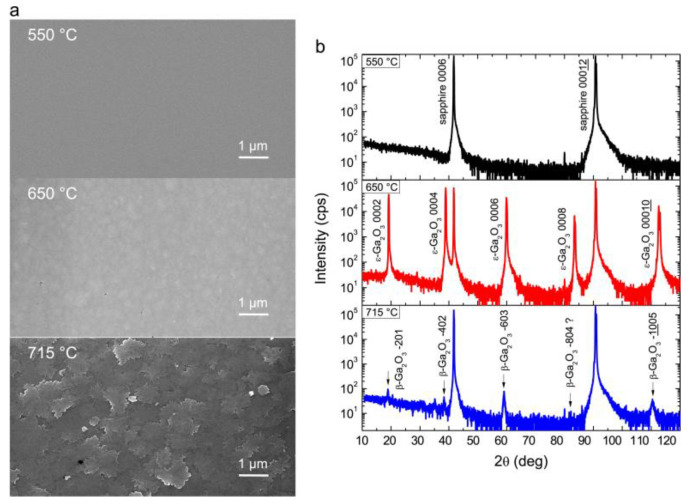
Ga_2_O_3_ samples grown at different temperatures on c-plane sapphire: (**a**) SEM images and (**b**) high-resolution XRD scan profiles. The thickness of the layers is 270, 200, and 250 nm, corresponding to T = 550, 650, and 715 °C, respectively (Reprinted with the permission of ref. [41]). Copyright 2016 under the terms of Elsevier publishing.

Meng et al. showed that the optimal growth rate of approximately 2.8 µm/h was achieved at 880 °C. At lower growth temperatures, such as 700 °C, the growth rate of β-Ga_2_O_3_ is constrained by the rate of the chemical reactions. Conversely, at higher temperatures, like 950 °C, the growth rate is hindered by gas-phase reactions and surface desorption, which become the limiting factors in achieving fast growth rates [109]. Mi et al. highlighted that a growth temperature of 650 °C is optimal for achieving the best crystallinity, with a full width at half maximum (FWHM) of 0.59°, for the growth of Ga_2_O_3_ on MgO (110) substrates [116]. Maintaining low-growth temperatures (down to 600 °C) can significantly reduce dopant diffusion, thereby preserving the sharpness of the doping profiles and enhancing the overall performance of devices [117]. Bosci et al. demonstrated that at a growth temperature of 550 °C, no crystalline layers were obtained, and the deposited films were amorphous with no X-ray peaks related to any gallium oxide phases, only showing α-Al_2_O_3_ reflections from the substrate [41]. At 715 °C, weak peaks corresponding to the (201) planes of β-Ga_2_O_3_ were observed, indicating poor crystalline quality with irregular surface morphology. The most notable results were achieved at an intermediate temperature of 650 °C, where films were smooth with very intense and narrow X-ray diffraction peaks, as indicated in Figure 11. The full width at half maximum (FWHM) of *ω-2θ* peaks was about 0.2°, and preliminary *ω*-scan measurements showed an FWHM of 0.3° for the (0006) peak.

#### 3.3.2. Effects of Reactor Pressure

**Figure 12:**
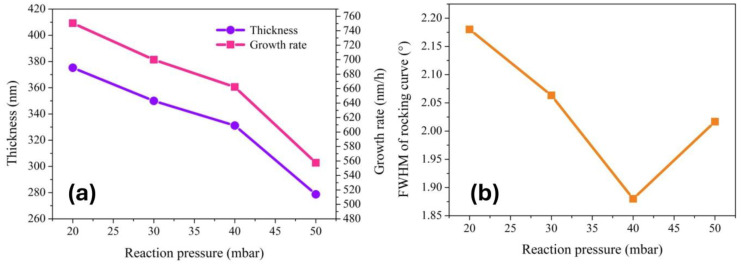
(**a**) The thickness and growth rates of the β-Ga_2_O_3_ films at different reaction pressures, (**b**) the average FWHMs of the rocking curves for the deposited β-Ga_2_O_3_ films (reproduced with the permission of ref. [114]). Copyright 2024 under the terms of Elsevier publishing.

A gradual decrease in the film-growth rate is observed when the chamber pressure increases from 20 to 100 Torr, which leads to higher silicon incorporation in the films [92]. This phenomenon is likely due to a decrease in precursor diffusion from the gas phase to the growth surface and an increase in the gas-phase reactions of the precursors. These findings underscore the critical influence of both temperature and pressure on the quality and characteristics of MOCVD-grown β-Ga_2_O_3_ films. Li et al. studied the effects of reaction pressure on the growth rate of films measured by VASE. As reaction pressure increased from 20 mbar to 50 mbar, the growth rate decreased from 750.5 nm/h to 557.5 nm/h, with a significant drop of 104.8 nm/h between 40 mbar and 50 mbar, as indicated in Figure 12a. The inverse relationship between reaction pressure and growth rate can be attributed to two main factors: 

Gas Phase Parasitic Reaction: When the organometallic source reacts with oxygen, represented as MO + O_2_ → (MO)x, most of the reaction products are carried out of the reaction chamber by the gas flow, leaving only a few solid particles within the films. The equilibrium Gibbs free energy for this gas-phase reaction is provided by [55]:

(10) $$-∆Ge=RTln\left(K\right)$$

(11) $$K=P^{-v}\exp\left(-\Phi \left(T\right)\right)$$

In these equations, ∆Ge represents the change in Gibbs free energy, *R* is the Boltzmann constant, *T* is the growth temperature, *K* is the equilibrium constant, v is the reaction coefficient, and $\Phi \left(T\right)$ is the function of the growth temperature. Higher reaction pressures enhance the gas-phase parasitic reaction, consuming more reactants [72,118].

Diffusion Coefficient of Reactants: The diffusion coefficient D for the MOCVD process is calculated as follows [55]:

(12) $$D=D_{0}\left(\frac{P_{0}}{P}\right){\left(T/T_{0}\right)}^{b}$$

Here, *D* is the diffusion coefficient, *D*_0_ is the diffusion coefficient at a reference pressure (*P*_0_), and the temperature is (*T*_0_). The value of “b” is about 1.82–1.88 for non-polar gases. *P* and *T* represent the reaction pressure and growth temperature used in the experiments. Increased reaction pressure reduces the material transport, thereby decreasing the growth rate [119,120]. Figure 12b displays the average FWHMs of the rocking curves, showing that as the reaction pressure increases from 20 mbar to 40 mbar, the FWHM decreases from 2.18° to 1.88°, indicating improved crystalline quality. This improvement is due to higher reaction pressure causing more reactants to be consumed by the gas-phase parasitic reaction, resulting in fewer atoms depositing on the substrates, which enhances atom migration. Additionally, the decreased gas-flow rate in the reaction chamber under higher pressure improves gas-flow uniformity. However, when the reaction pressure further increases to 50 mbar, the FWHM rises to 2.02°, reflecting a decline in crystalline quality. This decline is attributed to the significantly reduced growth rate, which enhances the gas-phase parasitic reaction, leading to more Ga_2_O_3_ nuclei forming and growing into islands on the substrate, thus degrading the crystalline quality.

**Figure 13:**
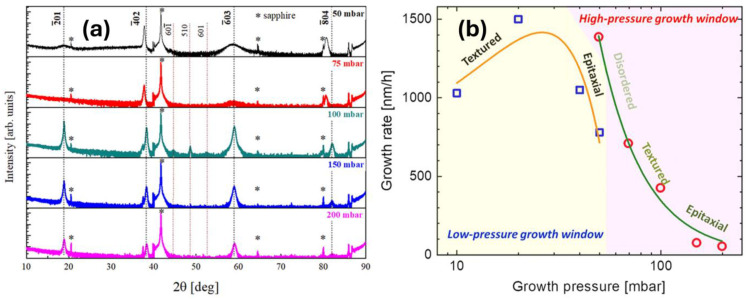
(**a**) The *2θ/θ* scans for β-Ga_2_O_3_ layers grown on c-plane sapphire at different pressures in Bragg–Brentano geometry. (**b**) The growth rate versus deposition pressure for two sets of samples. Red circles represent layers grown at high pressure, and blue squares represent layers grown at low pressure [121]. Copyright 2022 under the terms of CC BY 4.0.).

Figure 13a illustrates the growth characteristics of β-Ga_2_O_3_ layers on sapphire at various deposition pressures. The *2θ/θ* scans show that at pressures above 100 mbar, the layers exhibit a single (−201) orientation with prominent Bragg peaks, while at 100 mbar, additional undesirable crystallographic orientations appear. Below 100 mbar, the diffraction peaks broaden and decrease in intensity, indicating increased disorder. The growth rate versus pressure of Figure 13b reveals that at pressures below 100 mbar, growth rates can reach up to 1.4 μm/h due to rapid reagent reactions, but the layers become highly disordered and non-epitaxial. As pressure increases, the growth rate decreases significantly because of reduced atom diffusion and enhanced gas-phase reactions, which thicken the boundary layer. For pressures above 100 mbar, where single-orientation epitaxial layers are achieved, the growth rate drops to around 100 nm/h, which is too low for practical applications. To achieve high-quality, single-orientation β-Ga_2_O_3_ layers, a balance between pressure and growth rate is needed. High pressures help with epitaxial growth but slow down the growth rate, while lower pressures speed up growth but cause disordered films.

#### 3.3.3. Effects of Different Substrate Orientations on Homoepitaxy

**Figure 14:**
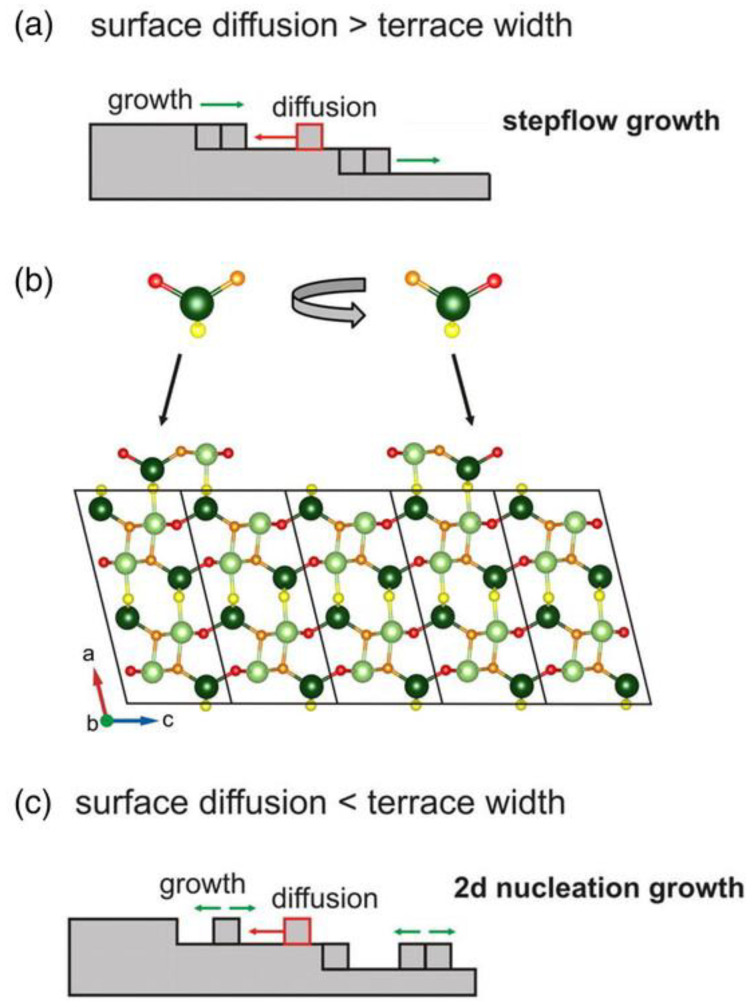
(**a**) Formation of 2D islands when the surface diffusion of adatoms exceeds the terrace width. (**b**) Double positioning resulting from 2D island formation. (**c**) Step-flow growth occurs when the surface diffusion of adatoms is less than the terrace width (reproduced with the permission of ref. [1]. Copyright 2022 under the terms of John Willey and Sons publishing.

The growth of β-Ga_2_O_3_ via MOCVD on (010) Ga_2_O_3_ substrates has been complemented by significant interest in the epitaxy on (100) Ga_2_O_3_ substrates. While direct on-axis growth on (100) substrates often results in incoherent boundaries, stacking faults, and impaired carrier transport, research has identified a solution to these challenges. Specifically, (100) substrates with off-axis angles ranging from 2° to 6° have been found to effectively suppress twinning domains, which are defects that occur due to mismatches in the crystal structure during growth. This strategic choice of off-axis substrate orientation is crucial for enhancing the quality of the epitaxial films [1]. The insights gained from the studies are vital for understanding both the surface dynamics and the chemical reaction kinetics essential for optimizing MOCVD growth on the (100) orientation of β-Ga_2_O_3_. Such findings not only help in achieving better structural integrity in the epitaxial layers but also in improving the electronic properties critical for device applications [104,122,123]. The (100) [124], (001), and (−201) [89,125] planes are low-symmetry planes that are easier to slice along the cleavage planes, making them commercially available on a larger scale. Epitaxial layers grown on these orientations are prone to twin boundary formation and stacking faults, which degrade electrical properties. The growth of the epitaxial layer can either follow the epitaxial orientation or form twinned lamellae, depending on the crystallographic orientation of the substrate and growth parameters. Defects like stacking faults and twin lamellae can be partially mitigated by controlling the diffusion length of adatoms, but monitoring adatom diffusion at subatomic levels at growth temperatures above 700 °C is challenging. Schewski et al. investigated how miscut orientation affects twin boundary defects [104]. They reported that β-Ga_2_O_3_ films grown on substrates with a miscut angle from 0° to 6° showed different behaviors. At miscut angles of 2° or less, growth proceeded by nucleation of 2D islands, resulting in stacking mismatch boundaries and twin lamellae. Increasing the miscut angle above 2° suppressed 2D island formation and initiated step-flow growth, leading to higher crystalline perfection. Substrates with higher miscut angles showed narrower terrace widths and uniform monolayer steps, facilitating faster adatom migration to terrace edges and monolayer nucleation. Conversely, substrates with lower miscut angles exhibited wider terraces and a mix of monolayer and bilayer steps, causing 2D island formation and double positioning. Adjusting growth conditions can enhance adatom surface diffusion, but mixed monolayer and bilayer steps cause nonuniform diffusion. The best epitaxial film morphology was observed at a miscut angle of 6° [104,123]. If adatom diffusion is shorter than the terrace width, adatoms may not always reach the terrace edge, leading to 2D island formation and twin lamellae, as illustrated in Figure 14a,b. Increasing the diffusion length of adatoms beyond the terrace width promotes step-flow growth and suppresses twin boundaries (Figure 14c). The direction of the miscut orientation also significantly affects the crystal quality of the epitaxial layer. Terraces on the (100) plane miscut towards the [00−1] direction are terminated by the (−201) plane, preventing bilayer formation and maintaining uniform terrace heights. Conversely, a miscut towards the [001] direction results in mixed monolayer and bilayer steps, leading to stacking mismatch boundaries. Bilayer steps have a step height of one unit cell, while monolayer steps have a step height of half a unit cell, causing nonuniform growth. Films grown on substrates with a 6° miscut towards the [001] direction showed poor electrical mobility of 10 cm^2^/V·s [123]. In contrast, films grown on substrates with a 6° miscut towards the [00−1] direction exhibited much higher electron mobility of 134 cm^2^/V·s [122], which improved to 144 cm^2^/V·s by optimizing the O_2_/Ga ratio and Ar carrier gas-flow rate [126].

#### 3.3.4. Effects of Different Gallium Precursors and Their Growth Rate

**Figure 15:**
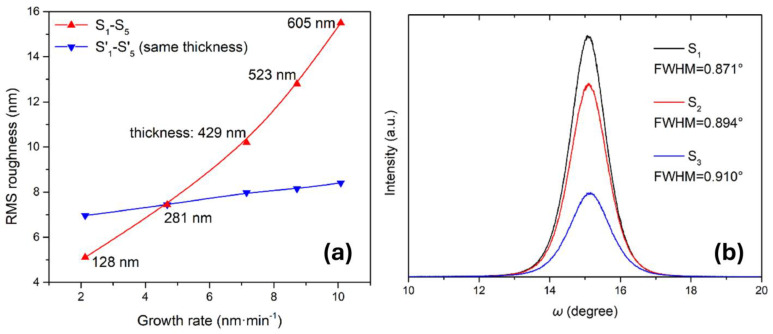
(**a**) Investigation of RMS roughness and crystalline quality of the Ga_2_O_3_ film as a function of film-growth rate. (**b**) A comparison of the XRD rocking curve for the different film thicknesses (reproduced with the permission of ref. [127]). Copyright 2022 under the terms of Elsevier publishing.

Wang et al. examined β-Ga_2_O_3_ films grown on SrTiO_3_ (100) substrates by MOCVD using TEGa precursor. At a low growth rate, the films were monocrystalline with an epitaxial relationship to the substrate. Increasing the growth rate from 2.1 to 10.1 nm/min increased surface roughness and decreased crystalline quality as indicated in Figure 15 [127].

**Figure 16:**
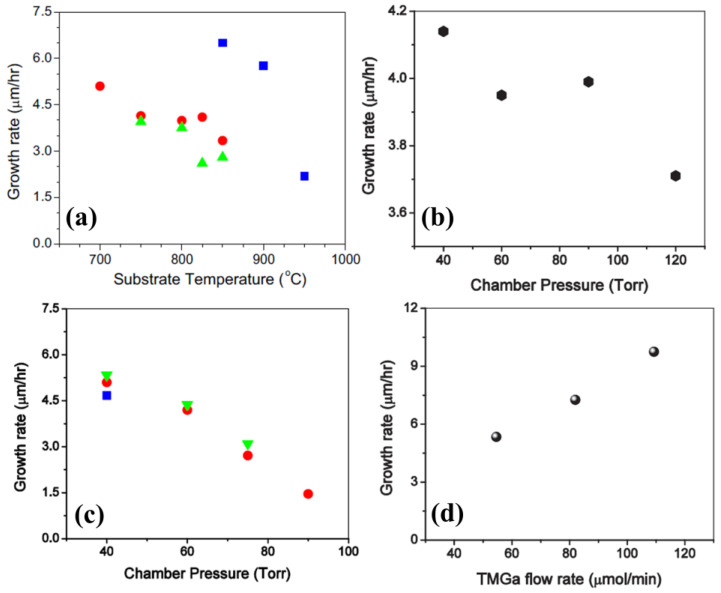
The growth rate of Ga_2_O_3_ films under various conditions: (**a**) versus substrate temperature at chamber pressures of 40 Torr (red), 60 Torr (green), and 75 Torr (blue), with TEGa flow of 50 µ·mol/min and oxygen flow of 300 sccm; (**b**) versus chamber pressure at 750 °C with TEGa flow of 50 µ·mol/min and oxygen flow of 300 sccm; (**c**) versus chamber pressure at 850 °C (red), 900 °C (green), and 950 °C (blue), with TMGa flow of ~55 µ·mol/min and oxygen flow of 800 sccm; (**d**) versus TMGa flow rate at 40 Torr, 900 °C, and oxygen flow of 800 sccm (Reproduced with the permission of ref. [91]). Copyright 2017 under the terms of Elsevier publishing.

A significant challenge in achieving high-quality MOCVD β-Ga_2_O_3_ epitaxy is managing the gas-phase reactions of precursors above the growth surface. Typically, growth rates for β-Ga_2_O_3_ range from 0.2 to 1.0 μm/h. These rates are primarily constrained by the gas-phase reactions, with Triethylgallium (TEGa) being the commonly used precursor. However, recent developments have shown that Trimethylgallium (TMGa) can also be used effectively as a precursor, offering high-quality homoepitaxial growth with rates potentially exceeding 3 μm/h, which is a notable improvement over those achieved with TEGa [108,109]. Figure 16a shows the growth rate of Ga_2_O_3_ thin films as a function of substrate temperature using TEGa as the source. The growth rate decreases with increasing substrate temperature due to the depletion of the TEGa source and parasitic reactions at higher temperatures. High substrate temperatures also promote the decomposition and desorption of Ga from the Ga_2_O_3_ surface, further reducing the growth rate. At the 40 Torr and 50 µ·mol/min TEGa flow rate, growth rates over 4 µm/h were achieved up to 825 °C, but increased chamber pressure reduced the growth rate as indicated in Figure 16b. Increasing the TEGa flow rate to 75 µ·mol/min achieved a growth rate of 6.5 µm/h at 850 °C and 40 Torr, but the growth rate dropped quickly at 950 °C due to source depletion and Ga desorption. Figure 16c shows that the growth rates of Ga_2_O_3_ thin films using the TMGa precursor decrease with increasing chamber pressure due to gas-phase reactions, similar to films grown with TEGa. However, unlike TEGa films, the growth rate for TMGa films is not significantly affected by the substrate temperature. This could be because TMGa requires high temperatures (>500 °C) to break down effectively, ensuring consistent Ga levels near the substrate. The higher oxygen flow (800 sccm) also helps maintain the growth rate by preventing Ga_2_O_3_ decomposition. Figure 16d shows that increasing the TMGa flow rate boosts the growth rate, reaching 9.8 µm/h at 109 µ·mol/min, the highest reported for Ga_2_O_3_ films by MOCVD. This indicates potential for large-scale production.

**Figure 17:**
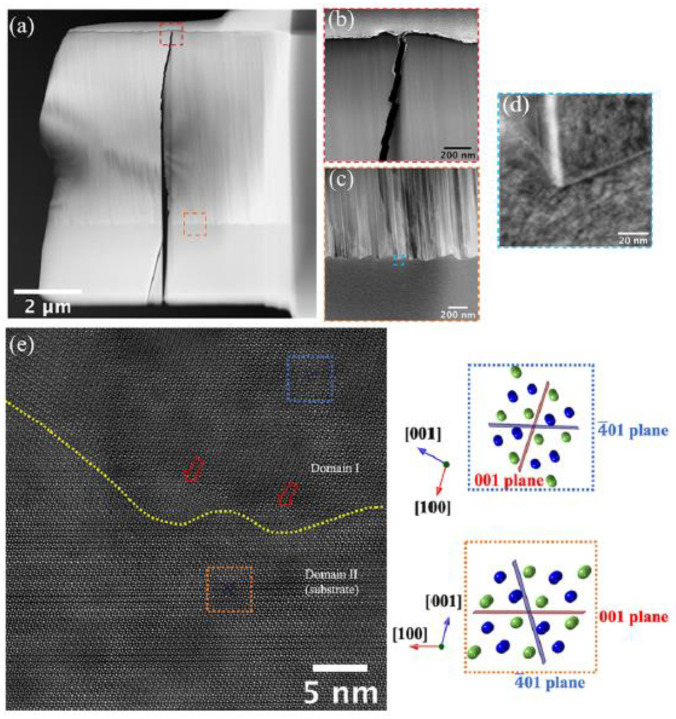
High-resolution STEM images of β-Ga_2_O_3_ films grown using TMGa on the (001) Sn-doped β-Ga_2_O_3_ substrate. The images display: (**a**) the presence of cracks in the film, (**b**) a zigzag pattern at the film-substrate interface, (**c**) a high-magnification view of the zigzag region, and (**d**) rotation domains within the crystal structure, (**e**) High-resolution STEM images of β-Ga_2_O_3_ film [128]. Copyright 2024 under the terms of ACS.

Figure 17 provides detailed insights into the formation of cracks in the film and substrate during the epitaxial crystal growth process. The cross-sectional STEM LAADF image (Figure 17a) reveals the presence of straight cracks running through both the film and substrate. These cracks are approximately 100 nm wide and over 8 μm deep. Upon closer examination of magnified regions near the surface (Figure 17b) and at the interface (Figure 17c), it is evident that the interfaces are nonuniform, suggesting a critical strain field has accumulated during growth. Further magnification (Figure 17d) and atomic scale details (Figure 17e) show that the film and substrate have different crystal structures, indicating a crystal rotation from the [001] direction to the [$\bar{4}$01] direction. This rotation alters the surface energies, leading to strain accumulation and stopping epitaxial growth along the [001] direction. Additionally, lattice reconstruction at the interface (highlighted by red arrows) causes lattice displacement, contributing to further strain accumulation. The presence of these strains and lattice displacements suggests that the choice of precursors might impact the stoichiometry of the film deposition. The interfacial lattice reconstruction observed in the images underscores the significant role of strain in the epitaxial growth process, indicating that managing these factors is crucial for achieving high-quality crystal growth [128]. 

#### 3.3.5. Effects of Different Substrates

While Metal-Organic Chemical Vapor Deposition (MOCVD) has been successful in achieving homoepitaxial growth of β-Ga_2_O_3_ thin films with superior structural and electrical characteristics, recent research has also explored heteroepitaxial growth of these films and their alloys on various substrates such as c-sapphire, GaAs, and silicon (Si) [121,129,130,131,132,133,134,135]. However, β-Ga_2_O_3_ epilayers grown hetero-epitaxially on these different, or “foreign”, substrates have not demonstrated the same satisfactory structural and electrical properties observed in homoepitaxial growth. For instance, when using on-axis c-plane sapphire as a substrate, β-Ga_2_O_3_ tends to grow with its ($\bar{2}$01) orientation aligned to the sapphire (0001) plane, but this alignment introduces rotational domains and lattice distortions [27]. Similar growth orientations of ($\bar{2}$01) β-Ga_2_O_3_ have been observed on GaAs substrates with varying orientations [20]. A particular study noted that MOCVD growth on silicon substrates resulted in amorphous Ga_2_O_3_ films, highlighting the critical influence of substrate selection on the crystalline structure and overall quality of the Ga_2_O_3_ epi films [23]. This suggests that while the MOCVD technique can produce high-quality β-Ga_2_O_3_ films, the choice of substrate significantly impacts the crystallinity of the epitaxial layer and functional properties.

Table 3 shows the optimized growth conditions of β-Ga_2_O_3_ films grown by both TMGa and TEGa precursors by various research groups to understand the effects of growth parameters on the crystalline quality of the films.

**Table 3:** Growth conditions for β-Ga_2_O_3_ films via MOCVD using TMGa and TEGa gallium precursors as reported by various research groups.

Research	Optimum Growth Temperature (°C)	Optimum Growth Pressure	Substrate	Growth Rate (µm/h)	Optimum FWHM	Optimized RMS Roughness (nm)	Hall Mobility	Deposition Time (min)	Carrier Gas and Flow Rate	Precursor O_2_ Flow Rate	Gallium Precursor Flow Rate
TMGa Precursor											
Li et al. (2020) [114]	750	40 mbar	(01) β-Ga_2_O_3_	0.62	21.6 (arcsec)	0.68	-	30	Ar (6N)-10 sccm	(5N)400 sccm	
Mi et al. (2012) [136]	650	20 Torr	MgO (110)	0.076	0.59°	-	-	360	N_2_ (9N)	50 sccm	2.7 µ·mol/min
Alema et al. (2017) [91]	900	40–120 Torr	(0001) C-sapphire	10	-	-	-	-	Ar (150 sccm)	800 sccm	50–109 µ·mol/min
Li et al. (2024) [137]	750	20–50 mbar	Sapphire (0001)	~0.1–0.75	2.18°–1.88°	4.6–6.7	-	30	-	(5N)	-
CaO et al. (2018) [117]	660	20 Torr	Epi-GaN/sapphire	0.25	0.5°	0.76		300	N_2_ (9N)	50 sccm	4.5 sccm
Gogova et. al., (2022) [121]	740 °C	10 mbar–200 mbar	α-Al_2_O_3_	1.4	-	-	-	60	Ar	515 mL/min	106 mol/min
Meng et al. (2022) [119]	950 °C	60 torr	Fe doped (010) Ga_2_O_3_	2.95	-	~1.23	190 cm^2^/V·s	60	-	800 sccm	58 µ·mol/min
Meng et al. (2024) [128]	TEGa: 880 °C; TMGa: 850–950 °C	60 torr	(001) Ga_2_O_3_	TEGa: 0.813; TMGa: 3.17	-	~2.56–10.2	85 cm^2^/V·s	30–180	Ar	TEGa: O_2_ = 400–1000 sccm; TMGa: O_2_ = 800 sccm	TEGa:12–31 µ·mol/min; TMGa: 58 µ·mol/min
Wagner et al. (2014) [138]	750–850 °C	5–100 mbar	(100) β-Ga_2_O_3_	0.18–0.48	-	6.5	-	30	Ar	O_2_: 200–1500 sccm; H_2_O: 400–1500 sccm	5 sccm
TEGa Precursor											
Seo et al. (2023) [122]	810 °C		(UID) GaN (002)/Al_2_O_3_	-	-	11.39	-	20	Ar	-	-
Tang et al. (2023) [139]	800–900 °C	10 KPa	β-Ga_2_O_3_	0.24–0.42	60 arcsec	1.3	51 cm^2^/V·s		N_2_	O_2_	68.2 µ·mol/min
Meng et al. (2022) [140]	880	60	on-axis (100) Ga_2_O_3_	0.66	60 arcsec	1.64	24 cm^2^/V·s		Shroud gas: Ar (300–2800 sccm)	O_2_: 800 sccm	VI/III ratio: 1150–2989
Chen et al. (2015) [118]	550	5000 Pa	GaAs (100)	0.89	-	6.41		60	Ar (5N)	(5N) 50 sccm	150 sccm
Alema et al. (2020) [141]	700 °C < T > 1100 °C	75 Torr < P > 500 Torr	Fe doped β-Ga_2_O_3_ (010)	-	41 arcsec	0.8	153 cm^2^/V s	-	Ar (6N)	N_2_O or oxygen (5N)	-
Zhang et al. (2019) [94]	-	-	β-Ga_2_O_3_ (010)	0.8	41.1 arcsec	0.8	176 cm^2^/V s	-	Ar (6N)	O_2_ (5N)	-
Yuea et. al. (2022) [142]	590 °C	25 torr	C-sapphire (0001)	0.775	1.71°	5.45	-	120	Ar (6N)	2000 sccm	350 sccm
Alema et al. (2020) [95]	-	-	β-Ga_2_O_3_ (010)	~1	~43 arcsec	0.8–13.4	>10^4^ cm^2^/V·s	-	Ar (6N)	O_2_ and H_2_O vapor source (0–250 ppm)	-
Chou et al. (2021) [126]	825 °C	25 mbar	β-Ga_2_O_3_ (100)	~1	30 arcsec	0.3–0.5	144 cm^2^/V·s	-	Ar (5N): 235 mmol/min	O_2_ (22 mmol/min)	73 µ·mol/min
Feng et al. (2019) [92]	880 °C	60 torr	Fe doped Ga_2_O_3_	0.65–0.7	-	~1.7	~140 cm^2^/V·s	-	-	-	VI/III ratio = 1150

### 3.4. Doping

Pure β-Ga_2_O_3_ should ideally be electrically insulating and optically transparent to visible light. However, it typically exhibits n-type conductivity even under undoped intrinsic (UID) conditions. The reasons behind this background n-type conductivity in β-Ga_2_O_3_ are still a subject of debate, with various potential factors contributing [143,144]. Several types of defects are known to influence the electrical properties of β-Ga_2_O_3_. These include gallium interstitials, hydrogen interstitials, vacancies (both gallium and oxygen), and other impurities such as hydrogenic dopants [145,146,147,148,149]. Initially, it was believed that the n-type conductivity of UID β-Ga_2_O_3_ was primarily due to oxygen vacancies [43,150]. However, further research, including studies by Varley et al., has shown that oxygen vacancies are deep donors with activation energies over 1 eV, which suggests that they cannot easily contribute to free carrier conduction at room temperature because they cannot be activated to release free electrons [43]. In contrast, certain impurities like hydrogen, silicon (Si), germanium (Ge), and tin (Sn) can act as shallow donors. Hydrogen typically occupies interstitial sites, whereas Si, Sn, and Ge substitute gallium on its lattice sites. These elements are commonly used as n-type dopants in β-Ga_2_O_3_ due to their effective contribution to electron conduction. Electron conduction in β-Ga_2_O_3_ is facilitated by the crystal’s conduction band minimum (CBM), which comprises unoccupied gallium-4s orbitals. These orbitals are spherically symmetric and have low energy, enhancing the material’s electron affinity and making n-type doping more feasible [151]. The introduction of n-type dopants elevates the Fermi level closer to the conduction band, increasing carrier concentration. However, dopants and other defects such as interstitials and vacancies can also act as scattering centers, which adversely affect electron mobility.

The dominant factor limiting electron mobility in β-Ga_2_O_3_ is polar optical phonon scattering within its monoclinic crystal lattice, typically restricting mobility to around 200 cm^2^/V·s. Additional mobility degradation results from scattering by ionized impurities and background defects. Growth conditions profoundly impact the formation of background doping and defects. For instance, oxygen-rich conditions tend to produce oxygen interstitials leading to a low background concentration of defects, while oxygen-poor conditions increase the likelihood of oxygen vacancies, which raise the background defect concentration [152,153]. Ga-rich or Ga-poor conditions similarly influence the formation of gallium self-interstitial defects or vacancies, affecting the material’s conductivity at room temperature. Impurities originating from growth precursors, like hydrogen and carbon, also play a significant role in defining the crystal properties of β-Ga_2_O_3_. Managing these defects is crucial for enhancing the electrical performance of power devices made from β-Ga_2_O_3_.

Lastly, p-type doping of β-Ga_2_O_3_ remains challenging due to the flat nature of its valence bands, formed by fully filled O-2p^6^ orbitals with low dispersion [154]. This structure leads to self-trapped holes with a large effective mass, resulting in very low hole mobility and limited options for effective shallow p-type dopants. The relationship between room temperature Hall mobility (cm^2^/V·s) and N-type carrier concentration (cm^3^) for various Ga_2_O_3_ thin films grown under different conditions are shown in ref. [55]. For instance, UCSB and Agnitron in 2018 used TEGa at 0.8 μm/h, UCSB in 2023 employed TEGa with a low-temperature buffer at 0.45 μm/h, and U. of Utah in 2020 used TEGa with a low temperature at 0.36 μm/h. Other studies include IKZ (Germany) in 2016 and in 2020 using TEGa at 0.3 μm/h, IKZ (Germany) in 2020 and in 2021 with different off-cuts, and TEGa at rates of 0.3 and 1.0 μm/h, respectively, and Agnitron ‘20 using TEGa with H_2_O assisted at 1.0 μm/h. Agnitron in 2020 and in 2021 also explored TMGa with rates ranging from 2 to 5 μm/h and N-doping. OSU in 2020 and in 2022 examined TEGa and TMGa with growth rates of 0.7 and ~3 μm/h, respectively. The data illustrate how different growth conditions and carrier concentrations impact the mobility of Ga_2_O_3_ thin films.

### 3.5. Summary and Future Prospects

The research on Metal-Organic Chemical Vapor Deposition (MOCVD) of β-Ga_2_O_3_ films has demonstrated significant progress in achieving high-quality epitaxial growth with different precursors, growth rates, and conditions. The use of Triethylgallium (TEGa) and Trimethylgallium (TMGa) as gallium precursors has enabled the optimization of growth rates and film quality. Notably, TMGa has shown potential for achieving higher growth rates compared to TEGa, with some reports indicating rates exceeding 3 μm/h. The primary challenge in the MOCVD growth of β-Ga_2_O_3_ is managing the gas-phase reactions of precursors above the growth surface, which affects the growth rate and film quality. This issue is further complicated by the impact of substrate temperature, chamber pressure, and precursor flow rates on the growth dynamics and resulting film properties. Future research should focus on refining the understanding of these parameters and their interplay to further enhance the growth rate without compromising material quality. Additionally, exploring novel reactor designs, such as the close-coupled showerhead system, can minimize gas-phase reactions and improve growth efficiency. Continued investigation into the effects of different substrate orientations and miscut angles is also crucial for optimizing epitaxial layer quality.

Overall, the advancements in MOCVD growth techniques for β-Ga_2_O_3_ highlight its potential for large-scale production of high-performance electronic and optoelectronic devices. Efforts to control defect formation, improve crystal quality, and enhance electron mobility will be key to realizing the full potential of β-Ga_2_O_3_-based technologies.

## 4. Halide Vapor Phase Epitaxy (HVPE)

### 4.1. Introduction

**Figure 18:**
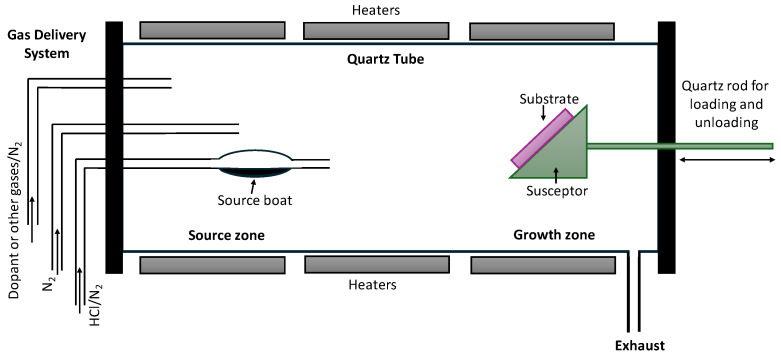
Schematic of a typical HVPE reactor system.

Halide vapor phase epitaxy (HVPE) is a high-growth-rate vapor phase epitaxy process used for growing high-quality epilayers and crystals with minimal incorporation of unintentional impurities [155]. This technique has been extensively employed in the development of III–V semiconductors [156]. For example, HVPE is one of the main methods of growing GaN free-standing bulk substrates [157]. Figure 18 illustrates a typical HVPE system used for growing nitrides or oxides. The system consists of a gas-delivery setup, a quartz tube, heaters, an exhaust, and a susceptor for mounting substrates. Based on gas-flow configurations, HVPE reactors can be categorized as either vertical or horizontal. Further details of reactor design can be found elsewhere [158]. A schematic of a typical horizontal hot-walled HVPE system, the most commonly used configuration, is provided (Figure 18). The reaction chamber is usually a quartz tube heated by a multizone oven (Heaters). The source zone is maintained at approximately 800–900 °C, while the growth zone, where precursors react on the substrate, is kept at around 1000–1100 °C. High-purity source metals (such as Ga, Al, or In) are placed on the source boat, and HCl or Cl_2_ gas is introduced to form metal chloride species. Subsequently, gases like NH_3_ or O_2_ are introduced in the growth zone to react with the metal chlorides and form the desired epilayer (e.g., GaN, AlN, or Ga_2_O_3_). HVPE is considered a near-equilibrium condensation process. Growth occurs almost immediately upon increasing the supersaturation of the vapor phase, causing a return to equilibrium and resulting in condensation. Decreasing supersaturation, such as by adding more HCl to the vapor phase, can reverse processes at the vapor-solid interface due to the volatility of chlorides. Consequently, HVPE can achieve fast growth rates (ranging from 1 to 100 µm/h) by increasing the material input of growth precursors [159]. The reactor design and experimental parameters in HVPE can be optimized so that gas-phase mass transport does not limit the growth of epilayers. Growth is predominantly controlled by the temperature-dependent kinetics of surface mechanisms, including adsorption and decomposition of precursors, desorption of by-products, and diffusion of ad-species. Additional details on growth thermodynamics can be found elsewhere [160].

**Figure 19:**
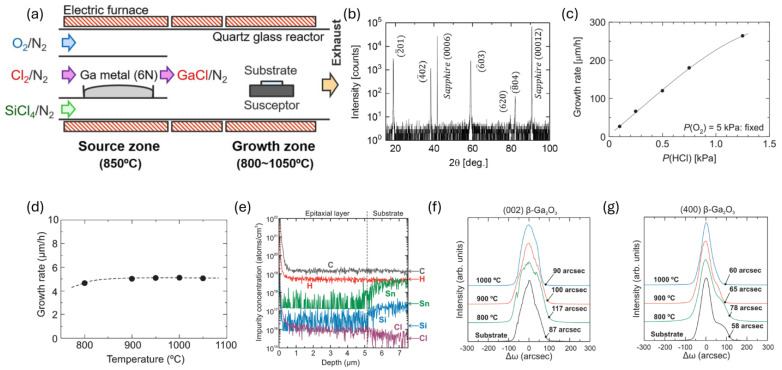
(**a**) Schematics of a Ga_2_O_3_ HVPE system. (**b**) X-ray diffraction (XRD) profile of a β-Ga_2_O_3_ epilayer grown on a sapphire (000) substrate with no off-angle. (**c**) Growth rate as a function of HCl partial pressure. (**d**) Growth rate of β-Ga_2_O_3_ on (001) β-Ga_2_O_3_ substrate. (**e**) Secondary ion mass spectrometry (SIMS) profile of an epilayer grown at 1000 °C. (**f**) Symmetric (002) and (**g**) skew-symmetric (400) XRD rocking curves. (**a**) Reproduced with permission from Ref. [66]. Copyright 2022 under the terms of CC BY 4.0. (**b**,**c**) Reproduced with permission from Ref. [164]. Copyright 2015 Elsevier. (**d**–**g**) Reproduced with permission from Ref. [39]. Copyright 2015 IOP publishing.

HVPE is a popular method for the rapid growth of thick, high-quality epitaxial Ga_2_O_3_ layers [66,161,162]. HVPE growth of Ga_2_O_3_ uses inorganic precursors, specifically Gallium (Ga) metal and Oxygen (O_2_). A typical Ga_2_O_3_ HVPE system, as shown in Figure 19a, consists of source and growth zones, with the source zone maintained at 850 °C and the growth zone ranging from 800–1050 °C. Nitrogen (N_2_), Helium (He), or Argon (Ar) serves as the carrier gas. GaCl is generated in the upstream region through the reaction of high-purity Ga metal (6N grade) with Clorene (Cl_2_). Since Cl_2_ reacts almost completely with Ga to form GaCl, the partial pressure of GaCl effectively doubles that of the input Cl_2_. Most of the time, Cl_2_ is replaced with HCl due to the high purity and ease of operation [161]. GaCl and O_2_ were introduced separately into the downstream growth zone (800–1050 °C). SiCl_4_ or SnCl_4_ can be introduced to the system when *n*-doped epilayers are needed. Nomura et al. [163] conducted a thermodynamic study and experimental demonstration of β-Ga_2_O_3_ homoepitaxy using HVPE. A stable growth at 1000 °C in an inert carrier gas requires a VI/III ratio above 1. The growth rate of Ga_2_O_3_ epilayers can range from a few µm/h to ~250 µm/h, depending on growth temperature, gas flow, and substrate [161]. However, HVPE-grown Ga_2_O_3_ films have significant surface roughness due to the high growth rate, needing an additional polishing step before device fabrication. Furthermore, the films contain Cl-induced impurities and defects resulting from the use of GaCl. This section mainly focuses on the HVPE growth of β-Ga_2_O_3_, with a brief exploration of its application to other Ga_2_O_3_ polymorphs, such as α- and ε-phases. The growth conditions, epilayer quality, mobility, and doping will be discussed.

### 4.2. Effects of Growth Conditions on Film Properties

Oshima et al. [164] demonstrated the growth of β-Ga_2_O_3_ on sapphire substrates, using off-angled sapphire (0001) substrates with angles ranging from 0° to 10°. The β-Ga_2_O_3_ films were grown directly on these substrates without any buffer layer to understand lattice mismatch. The XRD spectra, shown in Figure 19b, displayed a clear peak from the $\left(\bar{2}01\right)$ plane. The growth rates were around 250 μm/h with a fixed partial O_2_ pressure of 5 kPa, surpassing other Ga_2_O_3_ epitaxial techniques as shown in Figure 19c. At a zero off-cut angle, domain-like structures formed, but increasing the off-cut angle to 10° reduced these structures and favored in-plane growth. Murakami et al. later demonstrated the homoepitaxial growth of β-Ga_2_O_3_ on (001) β-Ga_2_O_3_ substrates over temperatures from 800 to 1050 °C [39]. They found the growth rate remained constant at approximately 5 μm/h at and above 900 °C (Figure 19d). At 1000 °C, with an input VI/III ratio of 10, growth was limited by the mass transportation of precursors, as predicted by thermodynamic analysis. Higher growth temperatures improved surface morphology and structural quality. SIMS and electrical analysis showed high-purity β-Ga_2_O_3_ layers with low-effective donor concentrations (Figure 19e). Impurity levels of C and H were below the background levels of the SIMS, and Sn and Si concentrations in the HVPE-grown homoepitaxial layer were lower than those in the substrate. Although Cl was detected in the layer, its concentration was low (about 10^16^ cm^−3^) and did not affect the carrier concentration. X-ray diffraction (XRD) rocking curves for the symmetric (002) (Figure 19f) and skew-symmetric (400) (Figure 19g) reflections showed full-width half maximums (FWHM) ranging from 117–90 and 78–60 arcsec, respectively, with increasing temperature. A Schottky barrier diode (SBD) was fabricated from a 12 μm-thick β-Ga_2_O_3_ epitaxial layer grown at 1000 °C on an *n*-type Sn-doped (001) β-Ga_2_O_3_ substrate. The C–V profile showed an almost constant capacitance regardless of the applied voltage, indicating complete depletion of the 12 μm-thick homoepitaxial layer, with depletion extending to the substrate. Thus, the *N_d_*–*N_a_* of the homoepitaxial layer was estimated to be <10^13^ cm^−3^, which is very low compared to other Ga_2_O_3_ growth techniques.

**Figure 20:**
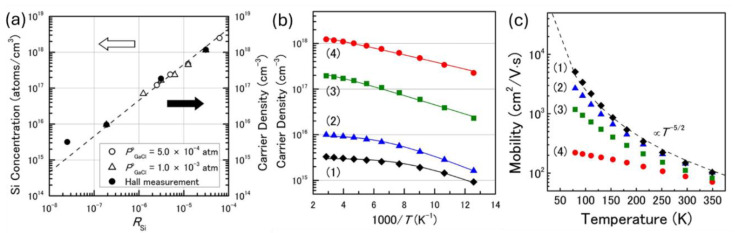
(**a**) Si carrier concentration measured by SIMS for two GaCl partial pressures and the carrier concentration measured by Hall measurements with respect to the ratio of GaCl to SiCl_4_ (*R_Si_*). Temperature-dependent (**b**) carrier density and (**c**) mobility for the respective samples, (1)–(4). (**c**) Reproduced with permission from Ref. [107]. Copyright 2018 Elsevier.

Goto et al. [107] demonstrated the growth of Si-doped homoepitaxial films on Fe-doped semi-insulating β-Ga_2_O_3_ (001) substrates using HVPE. The films were grown at a temperature of 1000 °C with an average growth rate of about 5 μm/h. The GaCl partial pressure was set to either 5.0 × 10^−4^ or 1.0 × 10^−3^ atm (Figure 20a), and the input VI/III ratio was maintained at 10. SiCl_4_ was introduced during the growth to achieve Si doping, with the partial pressure of SiCl_4_ controlled by the ratio of GaCl to SiCl_4_ (*R_Si_*) to regulate the doping concentration. To prevent Si dopant compensation by Fe diffusion from the substrate, an undoped layer (~1 μm) was grown between the Si-doped film and the substrate. The total thickness of the grown films, including the doped layer, was 7–9 μm. After growth, the Si dopant was activated by annealing at 1150 °C for 60 min in an N_2_ atmosphere. The surface roughness was addressed by polishing about 2 μm using chemical–mechanical polishing (CMP). Four samples with varying *R_Si_* ratios, resulting in doping concentrations from 10^15^ to 10^18^ cm^−3^, were grown (Figure 20b). SIMS analysis showed that the Si concentration varied proportionally with *R_Si_*, independent of the GaCl partial pressure, indicating that the Si dopant concentration is determined solely by *R_Si_*. This suggests nearly 100% activation of Si in the β-Ga_2_O_3_ films (Figure 20a). The activation energy was calculated to be between 17 and 46 meV. Ohmic contacts were formed using indium metal contacts for Hall measurements, performed in a Van der Pauw geometry between 80 and 350 K. The Si concentration and *n*-type carrier density of the samples are shown in Figure 20b. The temperature dependence of carrier density was measured for each sample (samples (1) to (4), with doping levels of 3 × 10^15^, 9 × 10^15^, 2 × 10 ^17^, and 1 × 10^18^ cm^−3^, respectively). The mobility varied with carrier density, being 149, 145, 111, and 88 cm^2^/V⋅s for samples (1)–(4) at room temperature. The temperature-dependent mobility was also measured, showing that in low-carrier-concentration films (Samples (1) and (2)), the mobility followed ∝ T^−5/2^ (Figure 20c), indicating optical phonon scattering dominance. Sample (1) reached ~5000 cm^2^/V⋅s at 80 K, suggesting the high quality of the film. In contrast, for the highly Si-doped film (sample (4)), the mobility increases with decreasing temperatures, indicating significant impurity scattering (∝ T^3/2^).

Apart from the above reports, Nikolaev et al. [165] demonstrated the growth of β-Ga_2_O_3_ on sapphire substrates by HVPE using metallic Ga, HCl, and dry air. They achieved high deposition rates ranging from 70 to 250 μm/h, with input VI/III ratios between 2 and 8. XRD and micro-Raman measurements confirmed that the films had a monoclinic β-Ga_2_O_3_ phase and were oriented along the $\left(\bar{2}01\right)$ plane. The FWHM of the $\left(\bar{2}01\right)$ peak decreased with increased GaCl flow, showing a minimum of 20 arcminutes. However, varying the GaCl flow also altered the deposition rate and the input VI/III ratio, making it unclear whether the narrowing of the rocking curve was due to the faster growth rate or the reduced VI/III ratio at higher HCl flows. Konishi et al. [166] investigated the homoepitaxial growth of β-Ga_2_O_3_ layers on Sn-doped (001) β-Ga_2_O_3_ substrates by HVPE using O_2_ or H_2_O as oxygen sources. Their results showed that the surfaces of layers grown with H_2_O were smoother than those grown with O_2_, although the growth rate with H_2_O was approximately half that of O_2_. Si contamination in HVPE reactors was reported due to the decomposition of the quartz glass (SiO_2_) reactor wall in the presence of hydrogen (H). Therefore, Si impurities with concentrations nearly equal to the effective donor concentration (2 × 10^16^ cm^−3^) when using H_2_O were observed. Leach et al. [167] reported the growth of β-Ga_2_O_3_ by HVPE on (010) β-Ga_2_O_3_ substrates. The substrates were prepared by removing surface damage and applying various miscuts to their surfaces. A clear difference in the morphology of the epilayer was observed between sufficiently polished and insufficiently polished substrates. Insufficiently polished substrates exhibited a large network of scratch-like features after growth, as seen by AFM, while sufficiently polished substrates were free of these features. Additionally, HVPE growth on (010) β-Ga_2_O_3_ substrates was strongly affected by the surface miscut. The epilayers on an on-axis substrate exhibited very rough morphology, while a substrate miscut by approximately 2° towards (100) showed a smooth morphology. Electrical properties were investigated on samples with unintentional and Si doping. The highest mobility of 123 cm^2^/V·s at a carrier concentration of 1.1 × 10^17^ cm^−3^ was achieved, whereas a low mobility of ~50 cm^2^/V·s was observed at a carrier concentration of 1.0 × 10^19^ cm^−3^. Xu et al. [168] demonstrated the heteroepitaxial growth of β-Ga_2_O_3_ on (0001) sapphire substrates with various off-angles towards the $<11\bar{2}0>$ direction. They used sapphire substrates with off-angles ranging from 0° to 10°. The off-angled sapphire substrates induced a transformation in the growth mode of β-Ga_2_O_3_ films, changing from six-fold in-plane rotational domain growth to single quadrilateral-domain growth. This significantly improved the crystal quality of the β-Ga_2_O_3_ films, with the films grown on approximately 7° off-angle sapphire showing the lowest FWHM (0.342°) and the flattest surface. It is important to note that HVPE growth of β-Ga_2_O_3_ tends to generate particles before reaching the substrate surface due to gas-phase reactions in the reactor. This can degrade the crystalline quality of the grown epilayer [169]. Furthermore, these particles can initiate abnormal growth, obstructing long-term high-quality epitaxial growth. To address this issue, Nitta et al. [169] introduced Trihalide Vapor Phase Epitaxy (THVPE) using GaCl_3_, which enables β-Ga_2_O_3_ growth without particle generation. GaCl_3_ can produce β-Ga_2_O_3_ with a relatively small free-energy change, avoiding particle generation compared to GaCl. GaCl_3_ was generated through a two-step reaction in the source zone, details of which can be found in Ref. [169]. However, the growth rate using this method is lower compared to conventional HVPE, reaching up to 32 μm/h on (001) β-Ga_2_O_3_ substrates by increasing the precursor partial pressure, with no particle generation. The FWHM of the (002) and (400) peaks are 50 and 28 arcseconds, respectively. Oshima et al. demonstrated HVPE growth of β-Ga_2_O_3_ on a native $\left(\bar{1}02\right)$ substrate, achieving growth rates as high as 23 μm/h. Rocking curves for the $\left(\bar{2}04\right)$ plane showed FWHM values of 27.5 and 25.9 arcseconds in the [010] and $\left[\bar{2}0\bar{1}\right]$ directions, respectively [170]. Additionally, $\left(\bar{4}01\right)$ diffraction in a skew-symmetric geometry exhibited a rocking curve FWHM of 26.6 arcseconds.

**Figure 21:**
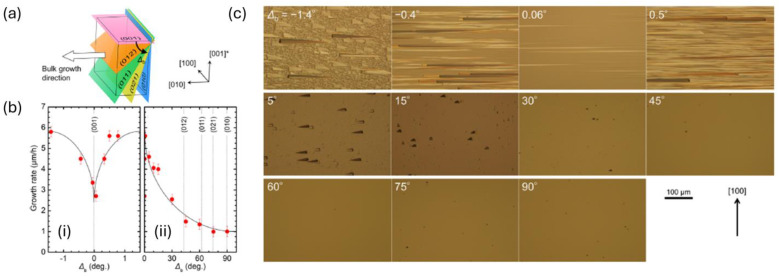
(**a**) Schematic of substrate orientations from (001) to (010) plane. (**b**) (**i**) The angle dependence of the growth rate near the (001) plane and (**ii**) from (001) to (010) plane. (**c**) Nomarski differential interference contrast (NDIC) microscopy images of epitaxial layers grown on various substrate angles. Reproduced with permission from Ref. [171]. Copyright 2022 AIP publishing.

Goto et al. [171] demonstrated the effect of substrate orientation on the homoepitaxial growth of β-Ga_2_O_3_ from the (001) plane. Initially, growth was performed near the (001) plane, varying the angle from −1.4° to 0.8°. The growth rate reached a minimum of ~2.7 μm/h near 0° and increased up to ~6 μm/h as the absolute value of the angle increased (Figure 21(b-i)). This behavior is attributed to the mirror symmetry of the β-Ga_2_O_3_ crystal structure (C2/m). Subsequently, substrates were cut at various angles from the (001) plane to the (010) plane from bulk crystals, covering angles from 0° to 90° (Figure 21a). Figure 21(b-ii) shows the growth rate for β-Ga_2_O_3_ on each substrate between (001) and (010). When the angle was greater than 0.8°, the growth rate decreased sharply, reaching ~1 μm/h as the angle increased. The grown surfaces were analyzed using Nomarski differential interference contrast (NDIC) microscopy (Figure 21c). On the nominally (001) substrate (0.06°), shallow streaky grooves formed along the [010] direction. As the angle increased, these grooves changed to triangular pits, with the directions of the pits reversing, owing to the crystal symmetry. As the angle increased further, the length of the triangular pits decreased, and when the angle exceeded 45°, the pits became difficult to recognize under NDIC microscopy. A pit-free surface was obtained at a 60° angle. When the angle reached 75° or greater, small dots were observed, which were identified as hillocks.

One challenge in growing thick epilayers using HVPE is the formation of parasitic particles in the chamber. These particles can negatively impact the quality of thick Ga_2_O_3_ layers by introducing defects such as stacking faults and etch pits [172]. The primary cause of this issue is attributed to the low gas-flow rate during HVPE growth. Although CMP cannot eliminate these defects, increasing the HCl flow rate can help minimize the formation of parasitic particles [170].

### 4.3. Selective Area Growth of Ga_2_O_3_ Using HVPE

**Figure 22:**
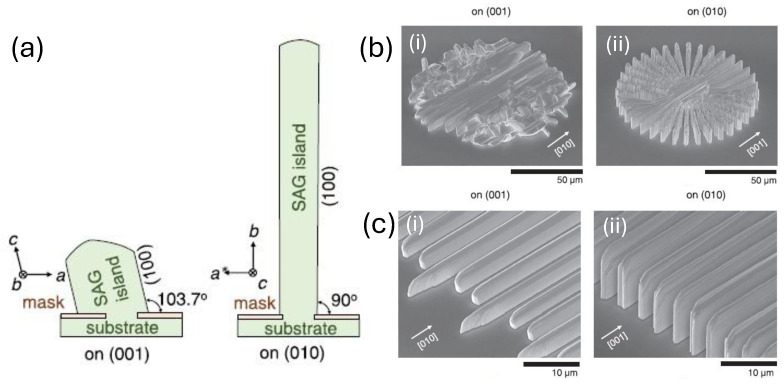
(**a**) Schematic cross-sections of the selective area growth (SAG) of β-Ga_2_O_3_ on (**a**) (001) and (010) substrates. (**b**) The scanning electron microscopy (SEM) images of radial-line patterns on (**i**) (001) and (**ii**) (010) orientations. (**c**) The SEM images of stripes patterns on (**i**) (001) and (**ii**) (010) orientations. Reproduced with permission from Ref. [173]. Copyright 2022 IOP Publishing.

Oshima et al. [173] showcased the selective area growth (SAG) of β-Ga_2_O_3_ using HCl-based HVPE and SiO_2_ masks. Patterned SiO_2_ masks were prepared on β-Ga_2_O_3_ (001) and (010) substrates, and Ga_2_O_3_ patterns/stripes were grown on both substrates as illustrated in Figure 22a. The inclination angle of the sidewalls of a stripe on the (001) substrate was approximately 104° from the substrate surface, consistent with the face angle between (001) and (100) planes. The lateral growth rates differed between the $\left[\bar{1}00\right]$ and [100] directions, with the rate along $\left[\bar{1}00\right]$ being 2–4 times faster than that along [100]. The grown patterns were characterized by scanning electron microscopy (SEM), as shown in Figure 22b,c. To understand the in-plane orientation dependence of SAG behavior, the shapes of the SAG β-Ga_2_O_3_ on radial-line patterns were grown (Figure 22b). Clear facet structures with smooth sidewalls were observed only when the patterned line directions were close to [010] and [001] on the (001) and (010) substrates, respectively. In other in-plane directions, growth behaviors differed significantly between the (001) and (010) substrates. On the (001) substrate, when the line direction deviated significantly from [010], polycrystalline grains with random orientations appeared instead of epitaxial growth (Figure 22(b-i)). On the (010) substrate, when the line direction deviated significantly from [001], the density of macro steps increased, leading to zig-zag-shaped sidewalls with no polycrystalline grains (Figure 22(b-ii)). The crystal anisotropy of β-Ga_2_O_3_ is responsible for these dramatic changes. Furthermore, Ga_2_O_3_ SAG stripes were grown on both planes as shown in Figure 22c. The difference in substrate planes was reflected in the heights and widths of the stripes, which are related to different vertical and lateral growth rates, respectively. The stripes on the (010) substrate (Figure 22(c-ii)) were much taller and narrower than those on the (001) substrate (Figure 22(b-i)). This work demonstrates that HVPE is capable of SAG to grow high aspect-ratio structures such as trenches and fins for β-Ga_2_O_3_ SBDs, Metal-oxide-semiconductor field effect transistors (MOSFETs), and fin-FETs.

### 4.4. Device Based on HVPE-Grown β-Ga_2_O_3_ Layers

Most high-voltage β-Ga_2_O_3_ devices, such as SBDs [174,175,176] and NiO/β-Ga_2_O_3_ p-n diodes [19,20,21], utilized HVPE to grow thick drift layers. Roy et al. [174] demonstrated a 687 V SBD with a 1.7 µm HVPE-grown drift layer (effective doping concentration of 9 × 10^16^ cm^−3^) and a surface breakdown field of 5.45 MV/cm, using high-k dielectric edge termination. Li et al. [175] demonstrated 2.44 kV Ga_2_O_3_ vertical trench SBDs with a net doping concentration of ~2 × 10^16^ cm^−3^ in the HVPE epitaxial layer, where the breakdown occurred at the trench bottom corner, sustaining a maximum electric field over 5 MV/cm. Dhara et al. [176] showcased trench SBDs with low-damage Ga etching, achieving a breakdown field greater than 5.10 MV/cm at a breakdown voltage of 1.45 kV, using a 10 µm-thick HVPE-grown epitaxial layer with a doping concentration of ∼5 × 10^16^ cm^−3^. Zhang et al. [177] demonstrated 5.1 kV and 8.2 kV NiO/β-Ga_2_O_3_ p-n diodes with 7.5 µm and 13 µm HVPE-grown drift layers, respectively. The net doping concentration of the epilayers was around 5–7 × 10^15^ cm^−3^, and the devices showcased breakdown fields of higher than 6 MV/cm. Gong et al. [178] demonstrated a field-plated NiO/β-Ga_2_O_3_ diode with a 7.7 µm-thick HVPE-grown drift layer. The non-field-plated device had a peak electric field of 6.7 MV/cm, while the field-plated device reduced the peak electric field to 4.9 MV/cm. Li et al. [179] demonstrated a 13.5 kV NiO/β-Ga_2_O_3_ diode with an 18 µm HVPE-grown drift layer, having a doping concentration of 8.8 × 10^15^ cm^−^^3^ and a high electric field of 7.5 MV/cm. The device consisted of an SiN_x_/SiO_2_ edge termination. The high-quality thick drift layers, primarily grown using HVPE, are crucial for achieving these high breakdown fields. The HVPE growth method enables a very low net electron concentration (~10^15^–10^16^ cm^−3^), enhancing high-voltage handling capability. Therefore, the HVPE growth of β-Ga_2_O_3_ is essential for the development of these high-performance devices.

### 4.5. Other Phases of Ga_2_O_3_ Growth Using HVPE

**Figure 23:**
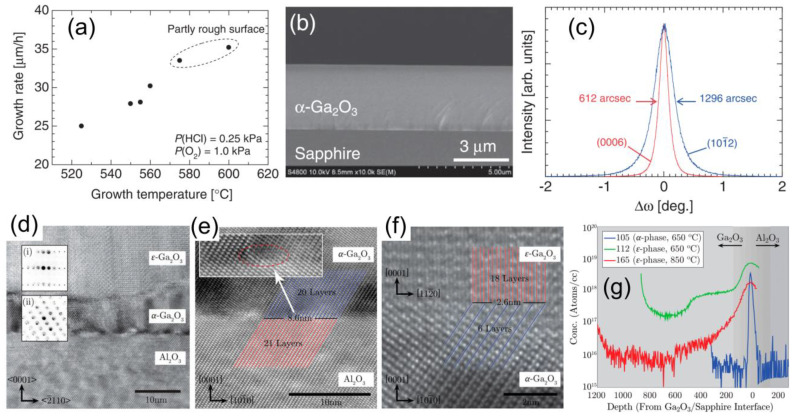
(**a**) Growth rate of α-Ga_2_O_3_ with temperature. (**b**) Cross-sectional image of an α-Ga_2_O_3_ layer grown on a sapphire (0001) substrate. (**c**) XRD rocking curve of (0006) and $\left(10\bar{1}2\right)$ peaks. (**d**) Cross-sectional HRTEM of α-/ε-Ga_2_O_3_ on c-plane sapphire. Diffraction corresponding to (**i**) ε-Ga_2_O_3_ $\left[\bar{1}100\right]$ and (**ii**) *α*-Ga_2_O_3_ $\left[11\bar{2}0\right]$. (**e**) Cross-section HRTEM of α-Ga_2_O_3_/c-sapphire interface. (**f**) Cross-section HRTEM of α-Ga_2_O_3_/ε-Ga_2_O_3_ interface. (**g**) SIMS profile of Cl− on three samples grown at different temperatures. (**a**–**c**) Reproduced with permission from Ref. [181]. Copyright 2015 IOP Publishing. (**d**–**g**) Reproduced with permission from Ref. [38]. Copyright 2018 under the terms of CC BY 4.0.

β-Ga_2_O_3_ is the thermodynamically most stable phase of Ga_2_O_3_ among the five different polymorphs α, β, γ, δ, and ε (or κ) [180]. Metastable rhombohedral α- and hexagonal ε-Ga_2_O_3_ phases have garnered recent interest due to their higher symmetry and simpler epitaxial relationships with c-plane sapphire [38]. The *α*- and ε-Ga_2_O_3_ were grown at temperatures ≤ 800 °C. Generally, the growth of β-Ga_2_O_3_ is above 850 °C. Oshima et al. demonstrated the initial HVPE growth of α-Ga_2_O_3_ on sapphire substrates, achieving twin-free films [181]. Figure 23a shows the growth temperature and rate, with rates reaching up to 35 µm/h at 600 °C. Despite the fast growth rate, the films developed a rough morphology above 575 °C. Figure 23b presents an SEM cross-section of a film grown at 550 °C with a thickness of 3.6 µm. The front surface showed a smooth surface in SEM and a mirror-like appearance to the naked eye. The XRD rocking curve profiles of the (0006) and $\left(10\bar{1}2\right)$ diffraction peaks, measured in symmetric and skew-symmetric geometries, respectively, for the film grown at 550 °C are shown in Figure 23c. The FWHM of the (0006) peak is 612 arcseconds, while the $\left(10\bar{1}2\right)$ peak is 1296 arcseconds. It was suggested that slower growth could improve the structural quality of HVPE epilayers and reduce FWHM.

Yao et al. [38] demonstrated the growth of α-, β-, and ε-phases of Ga_2_O_3_ using MOCVD and HVPE techniques. The β-phase was obtained by MOCVD, while α- and ε-phases were demonstrated by HVPE. The α- and ε-phases were identified using XRD and transmission electron microscopy (TEM). Figure 23d shows a cross-sectional HRTEM (high-resolution TEM) image of Ga_2_O_3_ on c-plane sapphire. After the growth of ~10 nm of α-phase, the epilayer transitioned to the ε-phase. Twinning in the ε -phase was observed due to its hexagonal sixfold symmetry and island nucleation growth mode. Figure 23e reveals a lattice offset at the sapphire/α-Ga_2_O_3_ interface, corresponding to the lattice mismatch between α-Ga_2_O_3_ and the c-plane sapphire. In a relaxed film, every 20 layers of α-Ga_2_O_3_ correspond to 21 layers of sapphire, generating a misfit dislocation every 8.6 nm. The film growth was postulated to follow a Stranski–Krastanov-like mode, starting with a semi-coherent layer of α-Ga_2_O_3_ on the sapphire substrate, followed by the island growth of ε-Ga_2_O_3_. Figure 23f shows the HRTEM image of ε-Ga_2_O_3_ on α-Ga_2_O_3_. The lattice spacing in the $\left[11\bar{2}0\right]$ direction of ε-Ga_2_O_3_ is 1.45 Å, and 18 layers of ε-Ga_2_O_3_ measure approximately 2.6 nm. The growth of α-/ε- phases can be explained as follows. The non-equilibrium growth during HVPE has a significantly faster growth rate, which did not allow adatoms time to rearrange into the thermodynamically stable β-phase. Each layer of α-Ga_2_O_3_ was constrained by the rapid deposition of subsequent layers. The sequential growth of α- and ε-Ga_2_O_3_ phases in HVPE-grown samples was believed to be associated with the presence of Cl, as revealed in SIMS investigations of samples grown under the conditions shown in Figure 23g. The presence of Cl had affected the formation of the ε-phase on top of the α-phase. More explanation and details about this can be found in Ref. [38].

Oshima et al. [182] demonstrated the epitaxial lateral overgrowth of α-Ga_2_O_3_ using HVPE with patterned SiO_2_ masks α-Ga_2_O_3_/sapphire (0001) templates. The α-Ga_2_O_3_ islands were selectively regrown on the mask windows in both vertical and lateral directions until they coalesced. Kawara et al. [183] further advanced this by demonstrating double-layered epitaxial lateral overgrowth of α-Ga_2_O_3_ using HVPE. Additionally, Oshima et al. [184] reported the growth of ε-Ga_2_O_3_ films on GaN (0001), AlN (0001), and β-Ga_2_O_3_ $\left(\bar{2}01\right)$ substrates by HVPE. Other phases of Ga_2_O_3_ have not been extensively studied, though there are some reports of their growth using HVPE [185,186,187,188]. In conclusion, HVPE offers a fast growth rate for Ga_2_O_3_ and can be used to grow thick Ga_2_O_3_ epilayers both heteroepitaxially and homoepitaxially, with intentional or unintentional doping and low background impurities. The high-quality thick drift layers are crucial components in Ga_2_O_3_ high-voltage devices. Various phases of Ga_2_O_3_ can be grown using HVPE by tuning the temperature, demonstrating the versatility of Ga_2_O_3_ growth by HVPE.

## 5. Mist Chemical Vapor Deposition (Mist-CVD)

### 5.1. Introduction

**Figure 24:**
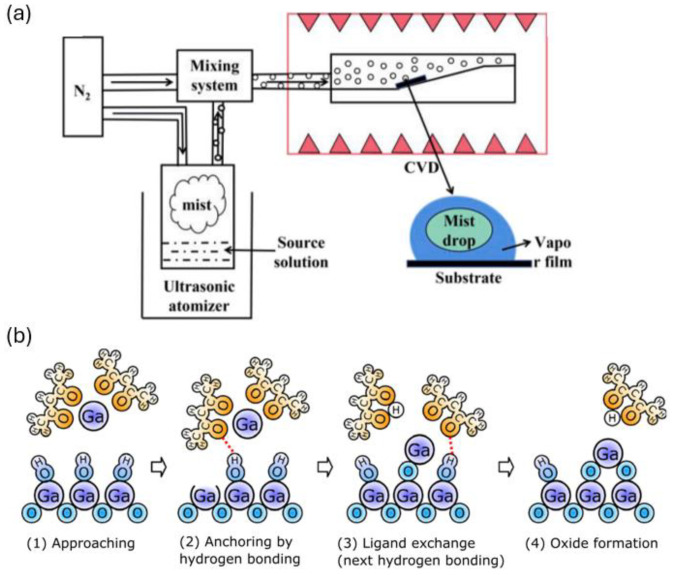
(**a**) Schematic of FC-type mist CVD system. (**b**) Proposed growth mechanism of Ga_2_O_3_ in mist-CVD. (**a**) Reproduced with permission from Ref. [196]. Copyright 2022 under the terms of Elsevier publishing. (**b**) Reproduced with permission from Ref. [194]. Copyright 2020 under the terms of MDPI publishing.

The mist chemical vapor deposition (mist-CVD) method has been widely utilized for growing metal oxides because it does not require vacuum operation. Furthermore, non-vacuum systems offer numerous benefits over vacuum systems, including lower environmental impact, reduced costs, simpler system configuration, and easier maintenance [189,190]. As shown in Figure 24a, a schematic diagram illustrates the experimental mist-CVD facilities. The gallium (Ga)-containing solution is converted into fine particles through high-frequency ultrasonic vibration. These particles are then carried into the reaction chamber by a carrier gas such as N_2_, O_2_, or clean air [189,191]. Furthermore, a dilution gas can homogenize the solution particles and enhance the final film quality. Typically, reaction chambers are designed with horizontal or inclined structures that can be sprayed by nozzles [192]. A vertical structure design was also reported [193].

The gallium source used in the growth process was an aqueous solution of gallium chloride. Before the source solutions were made, an undiluted aqueous solution of gallium chloride was prepared by dissolving Ga metal in 35.0–37.0% hydrochloric acid. The concentration of the undiluted Ga solution was [Ga^3+^] = 3:92 mol/L. Most of the Ga ions in this gallium chloride solution were expected to form the hexahydrate complex [Ga (H_2_O)_6_]^3+^ + 3Cl^−^. The acetylacetonate of Ga was controlled by the concentration of acetylacetone (Hacac). The acetylacetonate (acac) anions coordinate around Ga atoms in their enolate form. The possible ligand coordination structure is gallium acetylacetonate. The compositions of the source solutions employed were [G^3+^] = 0.02 mol/L and [acac^−^] = 0–0.06 mol/L. acac^−^ was introduced as NH_4_acac to shorten the complex formation time. After this, gallium acetylacetonate is used as a Ga source, dissolved in water with the assistance of hydrochloric acid (HCl). In this solution, it is expected to form [Ga(H_2_O)_6_]^3+^ + 3Cl^−^ complexes, as depicted in Figure 24b. Kazuyuki Uno et al. [194] suggested possible reactions leading to the formation of Ga_2_O_3_. The Ga acetylacetonate complex is positioned close to the surface, anchored by hydrogen bonding to surface hydroxyl groups. Subsequently, in Steps (2)–(4) of Figure 24b, a Ga-O bond is formed via a ligand exchange mechanism, with the ligand being acetylacetone (Hacac). It was specifically noted that oxygen (O_2_) originates from water and has less relation to O_2_ in the carrier gas. In the reaction chamber, the vapor-coated particles can adhere to the high-temperature substrate, where a chemical reaction can occur on the surface. In the mist-CVD process, as illustrated in the right-down corner of Figure 24a, droplets do not fully evaporate but are transferred onto the heated surface through a vapor film in the reaction chamber. This phenomenon, known as the Leidenfrost effect since 1756, involves droplets gaining energy from the heated surface, releasing the vapor into the air through mass flow. The Ga-containing solutes undergo chemical reactions on the substrate, transitioning stepwise to Ga_2_O_3_ growth. In the mist-CVD process, various growth conditions can impact both film quality and growth rate. The frequency of the atomization unit plays a significant role in determining the particle size. For instance, a 2.4 MHz atomization unit produces smaller particles compared to a 1.7 MHz unit. Since mist droplets initially have no velocity, their distribution tends to be floating and suspended. Therefore, precise control over particle flow is crucial for achieving the desired thin films. This reaction process continues until the droplets disappear completely.

Park et al. reported that microdroplets of approximately 2 μm in diameter were optimal for vertical structure mist-CVD [193]. Both numerical simulations and experiments have shown that a high deposition rate requires a low mist stream velocity and a substrate tilted at a high angle [195]. However, this poses a challenge in balancing the film-growth rate with carrier gas delivery. The design of temperature and gas-flow fields has attracted more research interest. The crystal phase of the substrate and other conditions also affects the reaction process. Despite the non-vacuum atmosphere in mist-CVD, complex structures can be prepared with atomic-level control simply by changing the precursor solution source. Gallium acetylacetonate is commonly used as a Ga source, dissolved in water with the assistance of hydrochloric acid (HCl). In this solution, it is expected to form [Ga (H_2_O)_6_]^3+^ + 3Cl^−^ complexes, as depicted in Figure 24b. Kazuyuki Uno et al. [194] suggested possible reactions leading to the formation of Ga_2_O_3_. The Ga acetylacetonate complex is positioned close to the surface, anchored by hydrogen bonding to surface hydroxyl groups. Subsequently, in Steps (2)–(4) of Figure 24b, a Ga-O bond is formed via a ligand-exchange mechanism, with the ligand being acetylacetone (Hacac). It was specifically noted that oxygen (O_2_) originates from water and has less relation to the O_2_ in the carrier gas.

### 5.2. Effects of Growth Conditions and Progress

#### 5.2.1. Epilayer Quality

**Figure 25:**
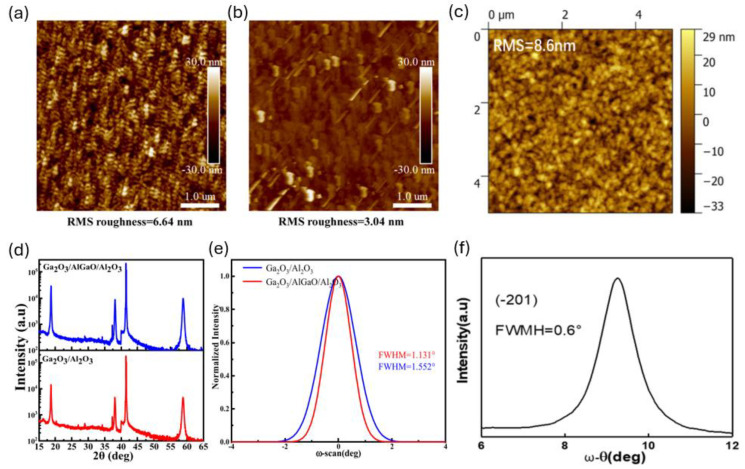
The results of AFM test of β-Ga_2_O_3_ thin film grown on (**a**) sapphire and (**b**) β-(Al_x_Ga_1−x_)_2_O_3_ (**c**) the GaN template. XRD test results of samples before and after using β-(Al_x_Ga_1−x_)_2_O_3_ (**d**) 2θ scan results and (**e**) rocking curve results. (**f**) rocking curve of the β-Ga_2_O_3_ (−201) diffraction peak. (**a**,**b**), (**d**,**e**) Reproduced with permission from Ref. [197]. Copyright 2021 under the terms of MDPI publishing. (**c**) Reproduced with permission from Ref. [196]. Copyright 2022 under the terms of Elsevier publishing.

**Figure 26:**
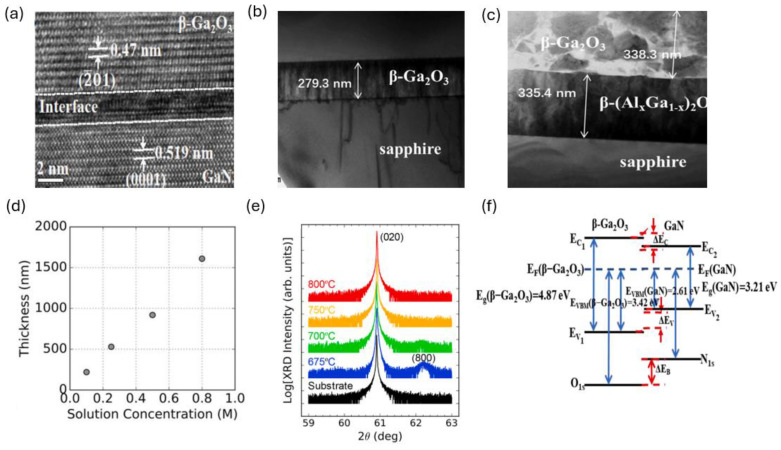
TEM image of the sample (**a**) β-Ga_2_O_3_/GaN interface (**b**) β-Ga_2_O_3_/sapphire, (**c**) TEM image of the sample of β-Ga_2_O_3_/β-(Al_x_Ga_1−x_)_2_O_3_/sapphire (**d**) film thicknesses as a function of solution concentration. (**e**) XRD of β-Ga_2_O_3_ thin films grown at temperatures 675 °C to 800 °C and a bare (010) β-Ga_2_O_3_ substrate. (**f**) The energy band structure of β-Ga_2_O_3_/GaN. (**a**,**f**) reproduced with permission from Ref. [196]. Copyright 2022 under the terms of Elsevier publishing. (**b**,**c**) Reproduced with permission from Ref. [197]. Copyright 2021 under the terms of MDPI publishing. (**d**,**e**) Reproduced with permission from Ref. [199]. Copyright 2021 under the terms of Elsevier publishing.

Cheng et.al used mist-CVD to grow β-Ga_2_O_3_ films epitaxially on sapphire substrates, and the β-(Al_x_Ga_1−x_)_2_O_3_ is used as the intermediate buffer layer to improve the β-Ga_2_O_3_ quality [197]. Figure 25a,b shows the atomic force microscopy (AFM) test results of the samples before and after using (Al_x_Ga_1−x_)_2_O_3_ as the intermediate buffer layer. The root mean square (RMS) roughness value of the sample before using the (Al_x_Ga_1−x_)_2_O_3_ buffer layer was calculated to be 6.64 nm, and after adding (Al_x_Ga_1−x_)_2_O_3_, the RMS was reduced to 3.04 nm. The 2θ scan and rocking curve test of XRD (X-ray diffraction) results are shown in Figure 25d,e. We could observe that after using (Al_x_Ga_1−x_)_2_O_3_ as the intermediate buffer layer, the intensity of the diffraction peaks increases, which means that the crystallinity of the film improves. The FWHM of the samples before and after using the β-(Al_x_Ga_1−x_)_2_O_3_ intermediate layer are 1.552° and 1.131°, respectively, which is significantly reduced and shows better crystallinity. Xu et.al reported the demonstration of lateral β-Ga_2_O_3_ Schottky barrier diodes (SBDs) fabricated on Fe-GaN/sapphire (0001) substrates by using the mist-CVD method for the first time [198]. The AFM result and rocking curve are shown in Figure 25c,f. The FWMH is 0.6° and the surface roughness is 8.6 nm. Figure 26a–c shows the cross-sectional transmission electron microscopy (TEM) image of β-Ga_2_O_3_/GaN, the β-Ga_2_O_3_ sample grown directly on a sapphire substrate, and three layers of β-Ga_2_O_3_/β-(Al_x_Ga_1−x_)_2_O_3_/sapphire, respectively. In Figure 26b, the thickness of the β-Ga_2_O_3_ layer is 279.3 nm. The growth rate of the β-Ga_2_O_3_ film is 9.31 nm/min, in which the thickness of the intermediate buffer layer is 335.4 nm, and the thickness of the β-Ga_2_O_3_ layer is 338.3 nm. It can be calculated that the growth rates of AlGaO and β-Ga_2_O_3_ are 11.18 nm/min and 11.28 nm/min [197], respectively. Thus, the use of the AlGaO intermediate layer can increase the growth rate of β-Ga_2_O_3_ (From 9.31 nm/min to 11.28 nm/min), and the band diagram of the Ga_2_O_3_/GaN heterostructure is shown in Figure 26f. Because it is easy to accumulate electrons at such a heterojunction surface, it can be used to make high-performance transistors. Nishinaka et al. [199] achieved the rapid homoepitaxial growth of β-Ga_2_O_3_ thin at a rate of 3.2 μm/h using a concentrated aqueous solution of the GaCl_3_ precursor. Figure 26d shows the film thickness as a function of solution concentration. The film thickness increased almost linearly with an increase in concentration. The XRD 2θ-ω scans of β-Ga_2_O_3_ homoepitaxial thin films grown at temperatures of 675–800 °C from 59° to 63° are shown in Figure 26e. A single (020) diffraction peak was observed in the patterns of β-Ga_2_O_3_ grown at higher temperatures of 750 °C and 800 °C. Conversely, the patterns of β-Ga_2_O_3_ grown at 675 °C and 700 °C exhibited small (800) peaks at a higher diffraction angle, which means growth at 750 °C and 800 °C could prevent the growth of β-Ga_2_O_3_ in other orientations.

#### 5.2.2. Carrier Transportation

**Figure 27:**
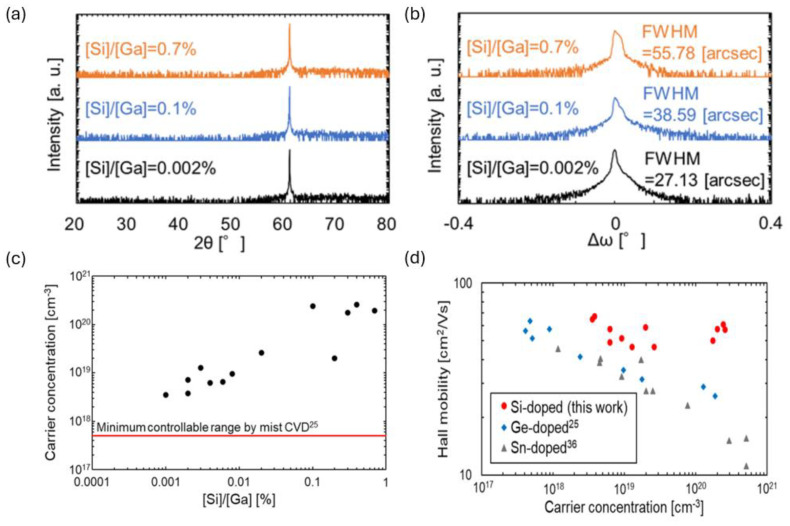
(**a**) XRD 2θ-ω scans of the (020) plane of Si-doped β-Ga_2_O_3_ thin films. (**b**) XRD rocking of the (020) plane of Si-doped β-Ga_2_O_3_ thin films and the FWHM values of the rocking curve peaks. (**c**) Carrier concentration of Si-doped β-Ga_2_O_3_ thin films obtained from room-temperature Hall effect measurement versus [Si]/[Ga] in the precursor solution. The red line shows the minimum controllable range of the carrier concentration in n-type doped β-Ga_2_O_3_ thin films prepared via mist CVD. (**d**) Relationship between the carrier concentration and Hall mobility of Si-doped β-Ga_2_O_3_ thin films grown by mist CVD compared with those of Ge-doped 25 and Sn-doped 36 thin films prepared by mist CVD. (**a**–**d**) Reproduced with permission from Ref. [202]. Copyright 2024 under the terms of AIP publishing.

Lee et al. [200] summarized the relationship between room-temperature mobility and carrier concentration in the Sn-doped β-Ga_2_O_3_ films. The mobility of the films monotonically decreased with increasing carrier concentration, suggesting dominant impurity scattering in the experimental region. The Sn-doped β-Ga_2_O_3_ film with a carrier concentration of about 1.0 × 10^18^ cm^−3^ showed a Hall mobility of 45 cm^2^ V^−1^ s^−1^ [200]. Ogawa et al. [201] reported the Ge-doped Mist-CVD growth β-Ga_2_O_3_. The carrier concentration increased as the concentration of the Ge precursor in the solution increased, indicating that the carrier concentration can be controlled by the Ge precursor concentration. The carrier concentration was controlled from 4.1 × 10^17^ to 1.9 × 10^20^ cm^−3^ by varying the Ge precursor concentration. Moreover, when using nitrogen and oxygen gases, the lower limit was approximately 5 × 10^17^ cm^−3^ and 0.9–6 × 10^18^ cm^−3^, respectively. The use of nitrogen gas resulted in fewer residual donors in the β-Ga_2_O_3_ thin films [201]. As illustrated in Figure 27a, the Si-doped β-Ga_2_O_3_ samples provided a single peak at ~60.9^○^, indicating that the thin films were homoepitaxially grown along the (010) plane. Figure 27b shows the XRD rocking curves of the different samples, along with their FWHM values. The slight right shoulder of the peaks in Figure 27b was observed, which shows that the lattice constant of β-Ga_2_O_3_ decreased with heavy Si doping, as smaller Si (IV) ions replaced Ga (III) ions [202]. As a result, the diffraction peaks shifted to the higher angle side.

Figure 27c illustrates the carrier concentration of the film against the Si concentration in the precursor solution used for mist CVD. The data reveal that the carrier concentration of the Si-doped β-Ga_2_O_3_ thin film can be controlled in the range of 3.85 × 10^18^ to 2.58 × 10^20^ cm^−3^ by varying the Si content in the precursor solution from 0.001% to 0.7% with respect to Ga. The highest carrier concentration achieved in this study is equivalent to that of Ge-doped β-Ga_2_O_3_ thin films grown by mist CVD. Figure 27d shows the relationship between the Hall mobility and carrier concentration of the Si-doped β-Ga_2_O_3_ thin films grown by mist CVD. For comparison, the results of Ge- and Sn-doped β-Ga_2_O_3_ thin films produced using mist CVD are also shown. Compared with the mobilities of the Sn-doped and Ge-doped β-Ga_2_O_3_ thin films, the homoepitaxial Si-doped β-Ga_2_O_3_ thin films grown in this study exhibit higher mobilities across the entire range of carrier concentration [202]. 

#### 5.2.3. Devices

**Figure 28:**
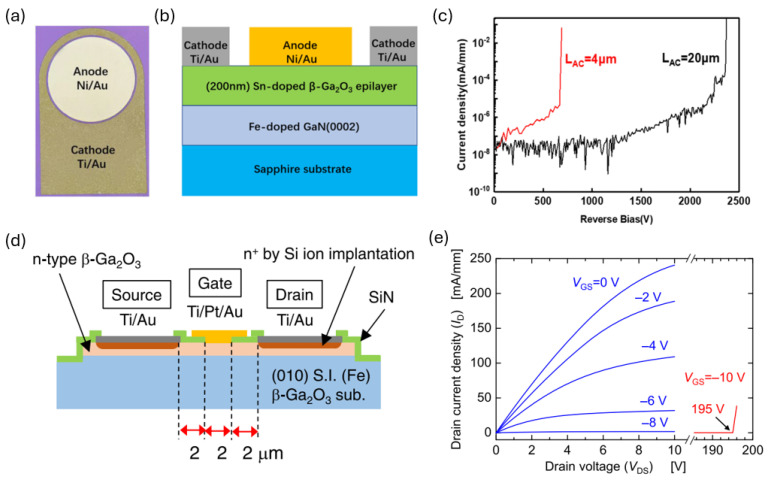
(**a**) Micrograph and (**b**) schematic cross-section of a β-Ga_2_O_3_ SBD. (**c**) Reverse I–V characteristics of β-Ga_2_O_3_ SBDs with LAC = 4 and 20 μm at room temperature. (**d**) Cross-sectional schematic image of the β-Ga_2_O_3_ MESFET structure. (**e**) Electrical properties of the fabricated β-Ga_2_O_3_ MESFET. Drain current density ID versus the drain-source voltage VDS for the different gate-source voltage VGS. (**a**–**c**) Reproduced with permission from Ref. [198]. Copyright 2021 under the terms of AIP publishing. (**d**,**e**) Reproduced with permission from Ref. [203]. Copyright 2023 under the terms of IOP publishing.

Yu et al. reported lateral β-Ga_2_O_3_ Schottky barrier diodes (SBDs) fabricated on Fe-GaN/sapphire (0001) substrates by using the mist chemical vapor deposition (mist CVD) method for the first time. The micrograph and schematic cross-section of the fabricated β-Ga_2_O_3_ SBD are shown in Figure 28a,b, respectively. Figure 28c shows the reverse V_br_ of the lateral β-Ga_2_O_3_ SBDs are as high as 560 and 2400 V for L_AC_ = 4 and 20 μm, respectively. Most of all, the measured high-breakdown voltages are based on basic and simple device structures, without any E-field reducing-device structure, such as a field plate [200].

Takane et al. [203] demonstrated an MESFET on a semi-insulating β-Ga_2_O_3_ (010) substrate by using mist CVD to grow the β-Ga_2_O_3_ channel layer. The cross-sectional schematic image of the MESFETs is shown in Figure 28d. The mobility and carrier concentration of the channel layer were 80 cm^2^ V^−1^ s^−1^ and 6.2 × 10^17^ cm^−3^, respectively. The device exhibited a pinch-off characteristic with a threshold gate voltage of −9 V, and the maximum drain current was 240 mA mm^−1^. The on-resistance was found to be R_ON_ = 30 Ω mm. The off-state breakdown under V_GS_ = −10 V occurred at V_DS_ = 195 V, as shown in Figure 28e. These devices’ performance suggests that mist CVD is a potential growth technology capable of providing low-cost devices in the future.

### 5.3. Summary

Overall, Mist-CVD has the unique advantage of the non-vacuum method and shows great potential in the Ga_2_O_3_ growth. By using an interlayer or leveraging the growth temperature, the qualities of the films have significantly improved. The doping concentration could be above 2 × 10^20^ cm^−3^, and the Si-doped films have achieved a high mobility of 57.2 cm^2^/V. The mist-CVD growth-based device’s performance suggests that it is a potential growth technology capable of providing low-cost devices in the future.

## 6. Pulsed Laser Deposition (PLD)

### 6.1. Introduction

**Figure 29:**
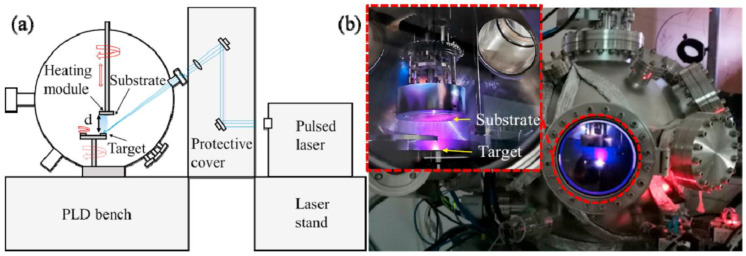
(**a**) A schematic diagram of the pulsed laser deposition system. (**b**) The plume of high-density plasma species during the film deposition process [207]. Copyright (2021) Elsevier.

Pulsed laser deposition (PLD) stands out among other methods of physical deposition due to several advantages: it is user-friendly, achieves high deposition rates, operates at low-growth temperatures, and is not restricted by the type of target material used. It also has already shown outstanding advantages in the epitaxial growth of oxide-thin films due to ultra-pure-growth environment, high-laser-energy density, and flexibly adjustable growth parameters [204,205,206]. During the deposition, a pulsed laser is used to vaporize and dissociate a target material. The resulting ejected species create a dense cloud that interacts with the laser pulse and surrounding gas, forming a highly excited plasma. This plasma, containing electrons, ions, and neutral species with a wide range of kinetic energies, eventually deposits onto a substrate to form a thin film [207]. Figure 29 shows us a schematic diagram of the PLD system and the film deposition process [205]. As a result, the thickness of films can be precisely controlled by controlling the number of laser shots used for ablation, as Figure 29 shows [6]. Based on all the advantages, PLD was widely utilized to grow Ga_2_O_3_ films, and various types of devices, such as photodetectors and Schottky barrier diodes, have been reported using PLD-grown Ga_2_O_3_ [208,209].

### 6.2. Effects of Growth Conditions on Film Properties

#### 6.2.1. Homoepitaxy

**Figure 30:**
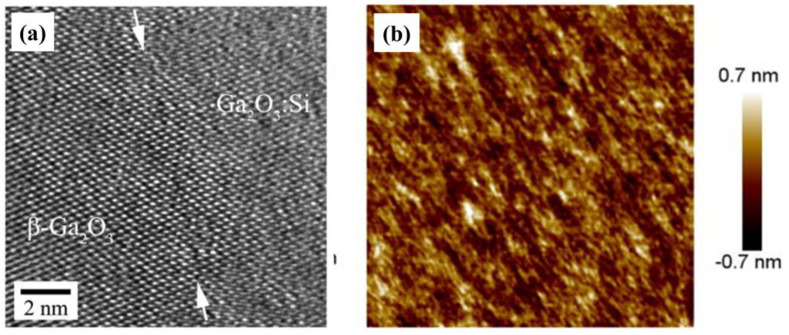
(**a**) TEM and (**b**) AFM images of Ga_2_O_3_: Si films [210]. The arrows indicate the interface. Copyright (2017) AIP publishing.

High-quality Ga_2_O_3_ thin films can be grown on Ga_2_O_3_ substrates by PLD directly. Yuxin et al. conducted the homo-epitaxial growth and obtained considerable smooth thin films with small RMS roughness between 0.43–1.43 nm under the best condition of 650–700 °C and 0.5 Pa [204]. The FWHMs of the β-Ga_2_O_3_ (400) peak of their films are 0.155°–0.193°. In Leedy et al.’s work, they deposited films on Ga_2_O_3_: Si substrates between 500 and 590 °C, and an observed [020] peak with an FWHM of 0.04°, which indicates the presence of pure phase [210]. Figure 30 shows the TEM and AFM of the Ga_2_O_3_: Si film. In this way, they obtained the high crystalline quality of the homoepitaxial layer, exhibiting a smooth, uniform morphology with a 0.2 nm RMS.

#### 6.2.2. Heteroepitaxy

**Figure 31:**
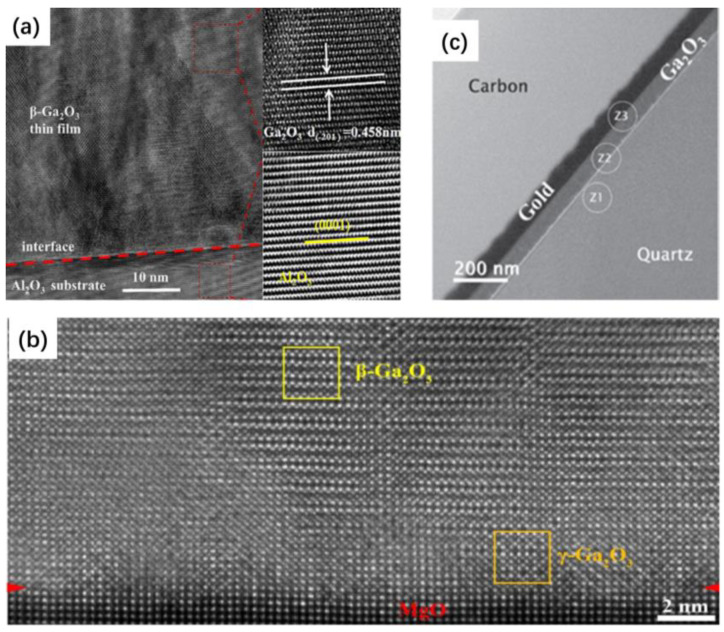
(**a**) HRTEM images of Ga_2_O_3_ thin film deposited on Al_2_O_3_ (0001) substrate. Redraw from [206]. (**b**) Atom-resolved HAADF image showing the domain boundaries in the β-Ga_2_O_3_ thin film and the γ-Ga_2_O_3_ formed in the interfacial region. Redraw from [207]. (**c**) Cross-section TEM images of Ga_2_O_3_ PLD thin film. Redraw from [195]. Copyright (2021) IOP Publishing.

C-plane (0001) of the Al_2_O_3_ is the most commonly used substrate for heteroepitaxial growth of Ga_2_O_3_ by PLD due to its hardness, and chemical stability. It also has a broad range of optical transparency and resistance to high temperatures and pressures [211]. Films with a full width at half maximum (FWHM) as narrow as 1.1° can be achieved through meticulous cleaning processes. As Figure 31a shows, the oxide film is not a perfect single crystal, but it is a β-Ga_2_O_3_ ($\bar{2}$01) 201)ǁAl_2_O_3_ (0001) epitaxial texture-dominated crystal film [204]. Hong et al. grew untralthinGa_2_O_3_ films on Al_2_O_3_ with different growth temperatures (400–1000 °C) and studied their structural, electrical, and optical properties [212]. The VBM of the Ga_2_O_3_ is measured to be about 2.8 eV, and the bulk Al_2_O_3_ is 2.26 eV with XPS. They determined the valence-band offset (VBO) is (−0.52) to (−0.74) eV consequently. In the process of RF plasma-assisted PLD, the energetic plasma stream will transport energy to ablated species, enhancing the reaction rate at the surface and adding surface mobility to the adatoms, which can lower the substrate temperature requirement for oriented growth. In this way, Congyu et al. grew Ga_2_O_3_ on Al_2_O_3_ at a low temperature (the temperature of the substrate is 200 °C) because the adatoms could obtain enough energy to occupy energy-favored locations in the Ga_2_O_3_ lattice for oriented crystallographic growth [213].

Besides Al_2_O_3_, MgO is another choice for heteroepitaxial growth because of its highly insulating nature and Eg of 7.8 eV, larger than that of β-Ga_2_O_3_ (Eg = 4.4–4.6 Ev). The epitaxial relationships between β-Ga_2_O_3_ and MgO are characterized by β-Ga_2_O_3_ (100) ǁ MgO (100) out-of-plane and β-Ga_2_O_3_ [001] ǁ MgO [011] in-plane. The lattice mismatch in this configuration is within ±3%. Ryo et al. demonstrated the growth of Si-doped Ga_2_O_3_ films on MgO (100) substrates using pulsed laser deposition (PLD) [214]. The films comprised β- and γ-phases, with twin domains observed specifically in the β-phase when grown at higher growth temperatures. The formation of these twin structures was attributed to the matching of second-nearest-neighbor oxygen atoms. The electronic properties of the films showed a clear dependence on the crystallinity of the β-phase. Notably, the crystallinity and conductivity were found to be better compared to films grown on α-Al_2_O_3_ (0001) substrates, highlighting the advantages of using MgO substrates for β-Ga_2_O_3_-based optoelectronic devices. Yiwen et al. investigated the fabrication of β-Ga_2_O_3_ thin films on MgO (100) substrates using varying growth temperatures and O_2_ partial pressures [185]. Across the temperature range of 500 to 800 °C, the full width at half maximum (FWHM) initially decreases and then increases, consistently measuring below 0.5°. This demonstrates that β-Ga_2_O_3_ thin films of high crystalline quality can be produced over a wide temperature range. However, as Figure 31b shows, there are many domain boundaries formed in β-Ga_2_O_3_ thin films, which may degrade the physical properties.

Other foreign substrates such as silicon and quartz have also been used. Berencén et al. explored crystalline β-Ga_2_O_3_ thin films on (100)- and (111)-oriented Si substrates and obtained polycrystalline with a deficit of oxygen atoms [215]. The mean surface roughness for β-Ga_2_O_3_ thin films deposited on (100)- and (111)-oriented Si substrates is found to be (2.8 ± 0.3) nm and (3.2 ± 0.3) nm, respectively. The crystal orientation of Silicon substrates does not affect the properties of the film. Vishal et al. reported pulsed laser-deposited (PLD) β-Ga_2_O_3_ films on the transparent quartz substrate [216]. They produced 200 nm-thick Ga_2_O_3_ films under temperatures in the range of 25–700 °C and found the films had variable microstructure and amorphous-to-nanocrystalline nature. The Ga_2_O_3_ films deposited at room temperature were amorphous; nanocrystalline Ga_2_O_3_ films could be realized at 700 °C. The roughness of the films is around 1–4 nm. In Figure 31c, the carbon, gold Ga_2_O_3_, and quartz layers are observed.

### 6.3. Growth Conditions

There are several key parameters in PLD growth conditions, including laser, substrate, temperature, and gas pressure. For the growth of Ga_2_O_3_ by PLD, the quality of the films is largely influenced by temperature and gas pressure.

#### 6.3.1. Temperature

Chen et al. deposited Ga_2_O_3_ films at different substrate temperatures on Al_2_O_3_ (0001) and analyzed the structural and morphological evolution [205]. They found the deposition rate decreased as the temperature increased from room temperature to 600 °C. This is likely due to increased surface energy of plasma species leading to re-evaporation or enhanced surface mobility and reduced sticking coefficient of Ga_2_O molecules. Additionally, the FWHM decreased with higher deposition temperatures, indicating improved crystal quality, which is also shown in grain sizes (from 23.9 nm to 31.1 nm). These results indicate that the thermal energy resulted in enhanced surface mobility by increasing the deposition temperature.

**Figure 32:**
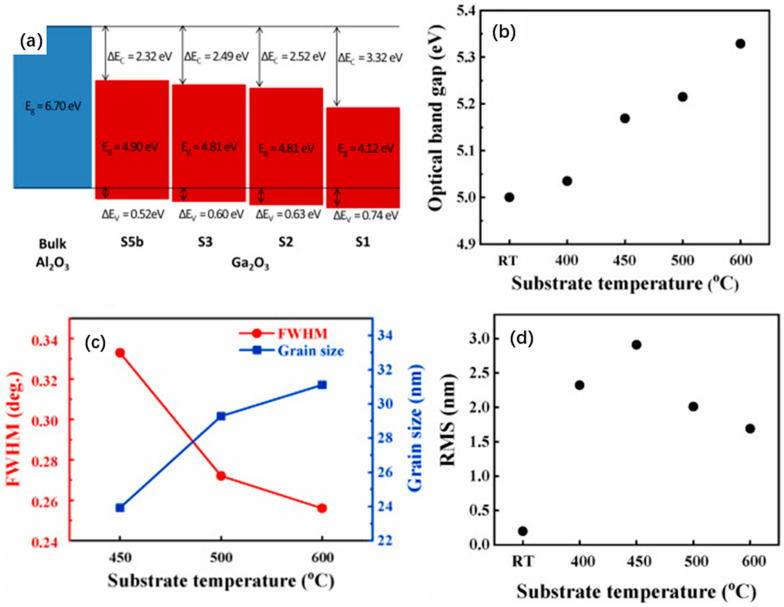
(**a**) The band alignment diagram of the β-Ga_2_O_3_/sapphire heterojunction. Redraw from [213]. The optical band gap (**b**), the variation of full width at half maximum (FWHM) and grain size (**c**) and the surface root-mean-square (RMS) roughness (**d**) for Ga_2_O_3_ films prepared at RT to 600 °C. Redraw from [205]. Copyright (2021) Elsevier.

Hong et al. found that in a higher temperature range from 400 to 1000 °C, the film will change from amorphous to single crystalline and then to polycrystalline with the rising deposition temperature [217]. This may be because increasing the films’ growth temperature can enhance the refractive index in the transparent area. Based on these results, they provide a further investigation [212]. They observed that as the growth temperature increases, the band gap widens, while both the valence band offset (VBO) and conduction band offset (CBO) decrease, as illustrated in Figure 32a. Their findings indicate that the β-Ga_2_O_3_/sapphire forms a Type II (staggered) heterostructure, with the band offsets ΔE_c_ and ΔE_v_ decreasing as the growth temperature rises, primarily due to reduced interfacial disorder. The effect of temperature on the growth of gallium oxide is shown in detail in Figure 32b–d [205].

#### 6.3.2. Oxygen Partial Pressure

**Figure 33:**
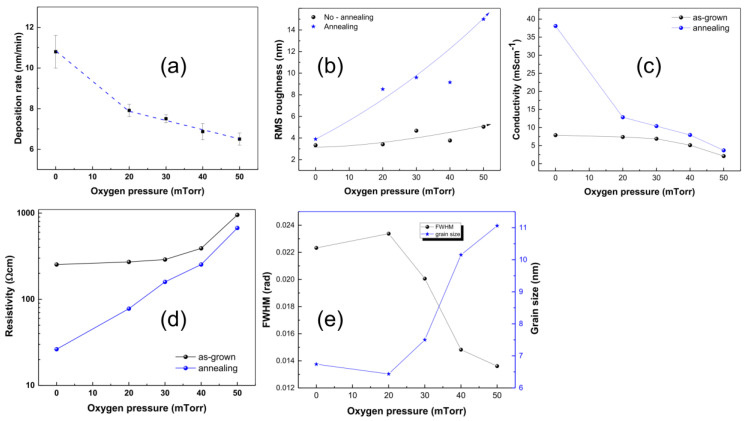
Evolution of the deposition rate (**a**), RMS roughness (**b**), conductivity (**c**), resistivity (**d**), and FWHM (**e**) of Ga_2_O_3_ thin films. Redraw from [218]. Copyright (2019) Elsevier.

Thi et al.’s work investigated the influence of oxygen pressure on the properties of Ga_2_O_3_ and observed the crystallinity of Ga_2_O_3_ enhanced with increasing oxygen pressure [218]. Figure 33 shows the dependence of several characteristics on oxygen pressure. After annealing, the full width at half maximum of the Ga_2_O_3_ peaks decreased, correlating with an increase in grain size from 6.76 nm to 11.25 nm. This suggests higher deposition pressure can improve plane texture and grain sizes. These results may be due to the reduced number of oxygen vacancies in high-oxygen pressure. A high rate was obtained without oxygen pressure, and it decreased with increased oxygen pressure. At a higher oxygen pressure, the ablated species are more likely to collide with oxygen molecules, causing the film-forming particles to thermally equilibrate and reduce their kinetic energy, thereby decreasing the growth rate. Because of the collision between deposited ad-atoms and oxygen molecules, atoms on substrates lack mobility to diffuse but form clusters, leading to an increase of RMS. Moreover, increasing the oxygen partial pressure can decrease the concentrations of VO and Gai in Ga_2_O_3_ films, leading to lower conductivity and higher resistivity of the films.

When grown with a relatively lower growth pressure, the most likely point defects in the film to develop are oxygen vacancies. However, growing the films at a higher oxygen partial pressure could result in other point defects, such as gallium vacancies [219]. Nicholas et al. grew films under the optimal growth pressure of 1 × 10^−3^ torr and minimized the point defects, which reduces the number of trapping centers that would capture the photogenerated carriers.

### 6.4. Doping

A variety of doped Ga_2_O_3_ with different dopants can be grown by PLD, which is essential for the fabrication of Ga_2_O_3_-based electronic and optoelectronic devices. Kevin et al. fabricated Si-doped Ga_2_O_3_ from Ga_2_O_3_ targets with 0.01–1 wt.% SiO_2_ [220]. They obtained widely varied concentrations (1.07 × 10^15^ to 1.37 × 10^20^ cm^−3^) under different oxygen pressures and peak mobility around 30 cm^2^/V s, which decreased at higher and lower pressures. Moreover, the conductivities of films from SiO2 targets (0.05 wt.% to 1 wt.%) increased with a deposition temperature up to 590 °C. However, films from 0.01 wt.% and 0.025 wt.% SiO_2_ targets showed notably lower conductivities. Sergiy et al. grew both Er-doped and Si and Er-co-doped Ga_2_O_3_ films by PLD, using highly pure Ga_2_O_3_, Ga_2_O_3_:1 wt% Si, and Er_2_O_3_ as targets [209]. They found that the carrier concentration of Si and Er co-doped Ga_2_O_3_ layer is lower than the Ga_2_O_3_: Si film, and Ga_2_O_3_: Si, Er film has the same result because of the degradation of crystallinity. Tao et al. investigated Si and Ta co-doped Ga_2_O_3_ films on Ga_2_O_3_, Al_2_O_3_, and quartz substrates [221]. They found that film on Ga_2_O_3_ substrate exhibits the best crystalline quality, and film quality on quartz substrate is the poorest. Compared to the quartz substrate, the carrier concentration of Si and Ta co-doped Ga_2_O_3_ films deposited on the Ga_2_O_3_ substrate is nearly two orders of magnitude higher. This can be attributed to a well-matched nucleating substrate, resulting in a low-defect, stress-free film.

### 6.5. Applications

**Figure 34:**
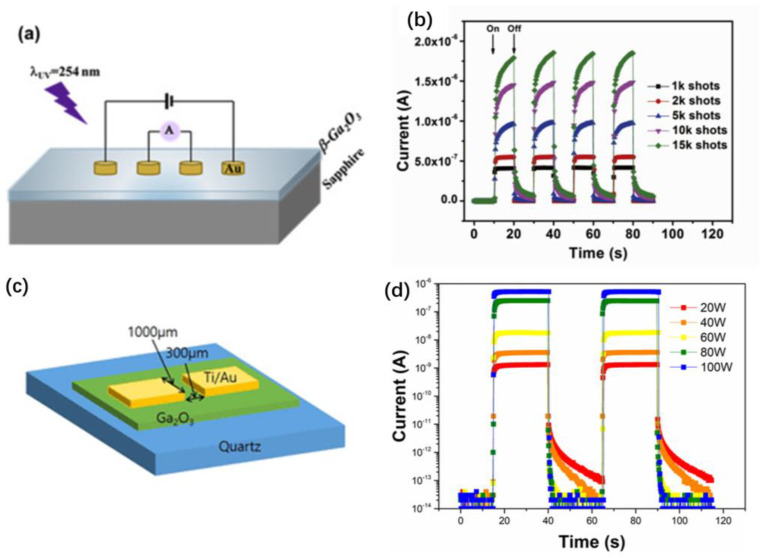
(**a**) schematic diagrams for time-dependent photocurrent. (**b**) Time-resolved photo response at a bias voltage of 30 V. Redraw from [209]. (**c**) Structure of metal-semiconductor–metal structure β-Ga_2_O_3_ photodetector. (**d**) Time-dependent photo-switching characteristics at plasma RF power of 20–100 W. Redraw from [222]. Copyright (2023) ACS Publications.

Ga_2_O_3_ films grown by PLD are mainly used for the fabrication of photodetectors. Hao et al. studied the influence of laser shots on the performance of photodetectors, and the structure of the device was shown in Figure 34a [208]. Films prepared with 2000, or fewer laser shots exhibited a high transmittance of approximately 80% for wavelengths above 300 nm. However, as the number of laser shots increased to 5000, 10,000, and 15,000, the transmittance began to decrease. They found that the thin films prepared with a lower number of laser shots (1 k and 2 k) had relatively smooth and flat surfaces, and the particle size showed a further increase when the number of laser shots increased to 5000 and more. As Figure 34b shows, for the films prepared with a larger number of laser shots, the dark current gradually increased, which can be due to the decrease in the sheet resistance with an increase in the thickness of the film. The 2 k-shot films demonstrated optimal photo response characteristics, boasting a high I_photo_/I_dark_ ratio (6.04 × 10^4^) and rapid response rates.

Taeyoung et al. studied the impact of varying the RF power of oxygen ions on photodetectors [222]. As the plasma power increased, the intensity of the O_2_ XPS peak decreased and the area ratio of O_2_/(O_1_ + O_2_) also decreased, which means that the effect of oxygen plasma reduced the densities of VO. The decrease in the VO concentration in β-Ga_2_O_3_ films leads to an increase in the photocurrent under UV illumination. Figure 34d shows the photocurrents of β-Ga_2_O_3_ photodetectors were markedly boosted with oxygen plasma treatment. The on/off ratio was increased to 106 in the 100 W device.

### 6.6. Summary and Future Prospects

The process of pulsed laser deposition (PLD) involves a series of intricate physical reactions. Essentially, a focused laser beam is directed at the surface of a solid target for a brief duration, generating an energetic plasma plume containing ions and atoms. This plume is then deposited onto a pre-heated substrate positioned in front of the target [223]. The growth and quality of the resulting film are generally influenced by various key factors, such as the choice of substrate, substrate temperature (Tsub), and the absolute and relative kinetic energies and arrival rates of the different components within the plasma. As technology has advanced, a variety of new lasers have come into use, ranging from mid-infrared (e.g., a CO_2_ laser at 10.6 µm) through near-infrared and visible (e.g., the Nd-YAG laser with fundamental and second harmonic outputs at 1064 nm and 532 nm, respectively) and down to ultraviolet (UV) wavelengths. Many current PLD studies utilize excimer lasers, which operate at different UV wavelengths (e.g., 308 nm (XeCl), 248 nm (KrF), 193 nm (ArF), and 157 nm (F2)) [224]. It is anticipated that there will be increased research on the PLD growth of Ga_2_O_3_ in the future.

## 7. Low-Pressure Chemical Vapor Deposition (LPCVD)

### 7.1. Introduction

**Figure 35:**
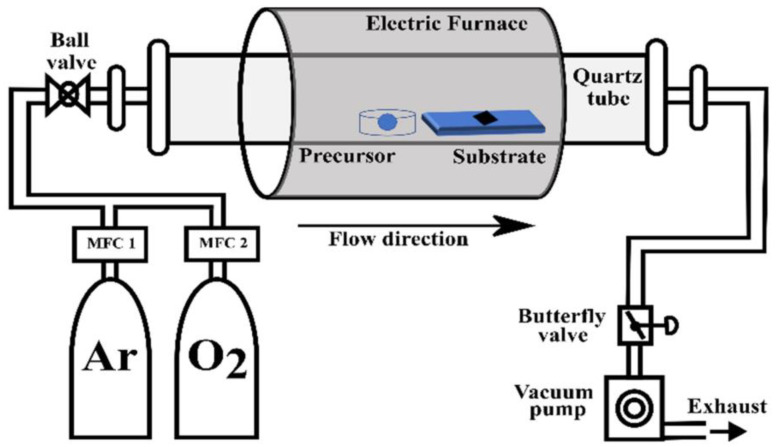
Schematic diagram of the LPCVD setup used for thin-film growth of Ga_2_O_3_ (reprinted from [226]).

Low-pressure chemical vapor deposition (LPCVD) is a widely used technique for the deposition of thin films, including β-Ga_2_O_3_. In LPCVD, high-purity gallium pieces and oxygen are used as precursors, while argon serves as the carrier gas [225]. The process involves placing gallium pellets in a multi-temperature zone furnace as shown in Figure 35. As the furnace heats up, the gallium vaporizes and is transported towards the substrate by the argon carrier gas. Simultaneously, oxygen gas is introduced above the substrate. The interaction between gallium vapor and oxygen results in the formation of Ga_2_O_3_, which crystallizes on the substrate surface. The by-product gases are subsequently expelled from the reaction chamber.

LPCVD offers several advantages in thin-film deposition. Firstly, it ensures high uniformity across the entire substrate, which is crucial for the production of consistent and reliable films [162]. The technique also facilitates the expulsion of source gases at high speeds, enabling the creation of abrupt heterojunctions, which are essential in various semiconductor applications. Moreover, operating under low pressure reduces the likelihood of premature gas-phase reactions, thereby allowing for higher growth rates. The reduced pressure environment also minimizes impurity incorporation at lower temperatures, enhancing film purity. LPCVD has been shown to produce films with defect levels comparable to or lower than those produced by metal-organic chemical vapor deposition (MOCVD) [227,228,229]. Additionally, LPCVD is cost-effective and scalable, making it suitable for the large-scale production of Ga_2_O_3_ films [230].

LPCVD can be utilized for both homoepitaxy and heteroepitaxy. Ga_2_O_3_ has been successfully grown on various substrates such as 4H-SiC, diamond, and sapphire [230,231,232,233,234,235,236]. Homoepitaxy is advantageous for creating vertical-device designs, while heteroepitaxy on sapphire is favored due to the relative cost-effectiveness of sapphire compared to Ga_2_O_3_. Other substrates can be selected based on their thermal properties and other design requirements, thereby offering versatility in device fabrication.

LPCVD-grown GaO_3_ has already been used to create several devices, including Schottky barrier diodes. These diodes have demonstrated impressive performance metrics, achieving a maximum electric field in the center of 4.2 MV/cm, a max electric field of 5.9 MV/cm at the edges, and an on-resistance (R_on_) of 3.9 m·Ω·cm^2^ [237]. These values indicate the potential of LPCVD-grown Ga_2_O_3_ for power and high-voltage applications, where high electric fields and low on-resistance are essential for efficient operation. Moreover, the LPCVD process has shown that even at high growth rates, the performance of Ga_2_O_3_ devices remains robust, with one study demonstrating devices that were grown at a rate of 16 µm/h maintained a good forward and reverse IV performance, demonstrating the capability of LPCVD to produce high-quality films rapidly without compromising device functionality [238]. This high growth rate is particularly advantageous for industrial-scale production, where throughput is a critical factor. In addition to electronic devices, LPCVD-grown Ga_2_O_3_ has also been utilized in optoelectronic applications, such as solar-blind detectors and deep ultraviolet (UV) detectors [239,240].

### 7.2. LPCVD Growth of β-Ga_2_O_3_

#### 7.2.1. Growth on Sapphire

**Figure 36:**
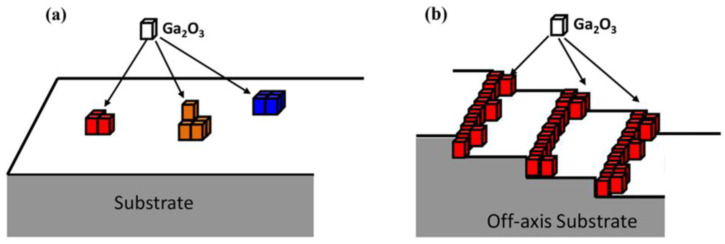
A comparison between the LPCVD growth of a Ga_2_O_3_ on C-plane sapphire (**a**) without an off-axis angle and (**b**) with an off-axis angle (reprinted from [243]).

**Figure 37:**
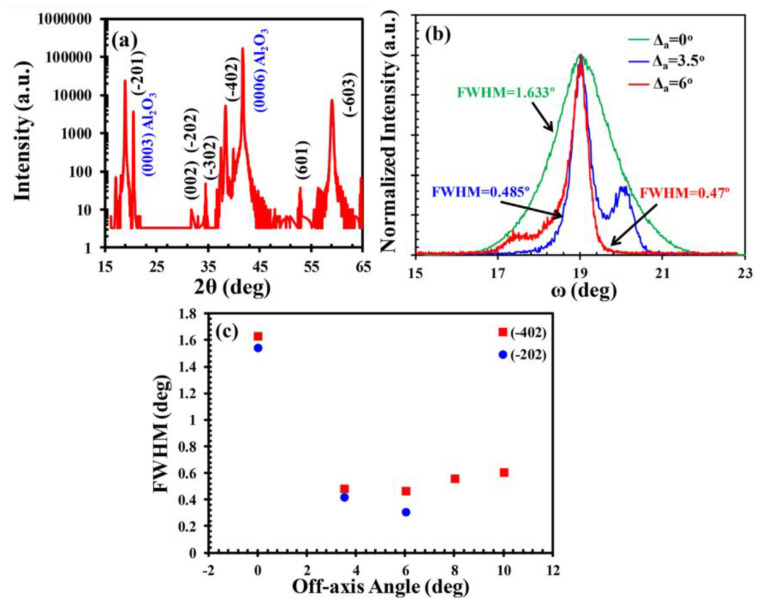
(**a**) The XRD scan of the Ga_2_O_3_ film with no off-angle. (**b**) A comparison of how the XRD reflection changes at the (−402) plane. (**c**) The change in the FWHM of the XRD with changing off-axis angle. (reprinted from ([243]).

The use of off-angled substrates is a common practice in crystal growth due to the enhanced quality of the resulting thin films [241,242,243]. The off-axis orientation introduces step-like structures on the substrate surface, which act as preferential binding sites for Ga adatoms [243]. These binding sites promote step-flow growth and reduce random nucleation on the surface, and the incorporation kinetics of the steps and the diffusion of ad atoms were some of the most important principles to consider for this type of growth. The size of the step terrace is critical, as it must match the diffusion length of Ga atoms to maintain consistent surface morphology. If the step size is too large, it can cause the formation of island growth. The diffusion length is influenced by the growth parameters, while the step size is determined by the off-axis angle. The off-axis angle that produced the best-quality thin films on sapphire was found to be 6° for many studies [243]. Without an off-axis angle, there are no preferential sites for Ga adatom binding, leading to inferior film quality. It illustrates how the growth is affected in Figure 36a with no off-axis angle and Figure 36b with an off-axis angle. The increased quality of the off-axis growth can increase the thermal conductivity of the Ga_2_O_3_ film, and it has been reported that sapphire has increased from 9 to 17 W/mK [133]. However, the thermal boundary resistance between the substrate and the epitaxial Ga_2_O_3_ has been shown to increase with the off-angle [133]. It also results in an increase in electron mobility, with one study reporting a nine-fold increase in the mobility between the no-offset angle and the 6°-offset angle [244]. The offset also resulted in a reduction of the FWHM. Figure 37 shows this reduction in the FWHM with an off-angle (a) by first showing the XRD of the film with no off-angle, then with (b) showing how the FWHM reduces for the reflection at (−402) plane, and (c) shows the change in all the XRD peaks due to the off-angle.

The quality of Ga_2_O_3_ films on sapphire substrates is typically assessed using metrics such as surface roughness, electron mobility, and the FWHM of the XRD rocking curve. The growth rate is also an important consideration, as it affects the time required to achieve the desired film thickness. Key LPCVD parameters that influence these metrics include growth temperature, growth pressure, the distance between the substrate and the crucible, and the oxygen-flow rate [244]. The off-axis angle and the crystalline plane of the substrate also play significant roles. The c-plane of sapphire provides the best results for (−201) Ga_2_O_3_ due to its lattice compatibility [225].

High-temperature LPCVD (HTLPCVD) has achieved remarkable results, with maximum electron mobility of 126 cm^2^/V·s, an XRD rocking curve FWHM of 0.09°, a root mean square (RMS) roughness of 1.82 nm, and a growth rate of 31 µm/h [229,244,245,246]. However, above approximately 925 °C, the electron mobility and other metrics tend to degrade [232,245]. Nevertheless, studies have shown that increasing the oxygen flow rate can maintain high electron mobilities at temperatures as high as 1050 °C [246]. This study also showed that by increasing the oxygen-flow rate at higher temperatures, the growth rate could also be increased. This is because, at higher temperatures, the evaporation rate of Ga will increase, and an increased oxygen flow rate will maintain the Ga/O balance [246]. Post-growth annealing has also been shown to improve the FWHM of the XRD rocking curve [247].

A critical variable that heavily affects the growth rate is the distance between the substrate and the Ga precursor [246]. This happens because the increase in the distance between Ga and the substrate results in a reduction in the concentration of the reactive species and a decreased rate of the gas-phase reaction, which decreases the growth rate of the film [244]. The growth rate decreases as a decaying exponential with increased distance between the Ga and the substrate.

**Figure 38:**
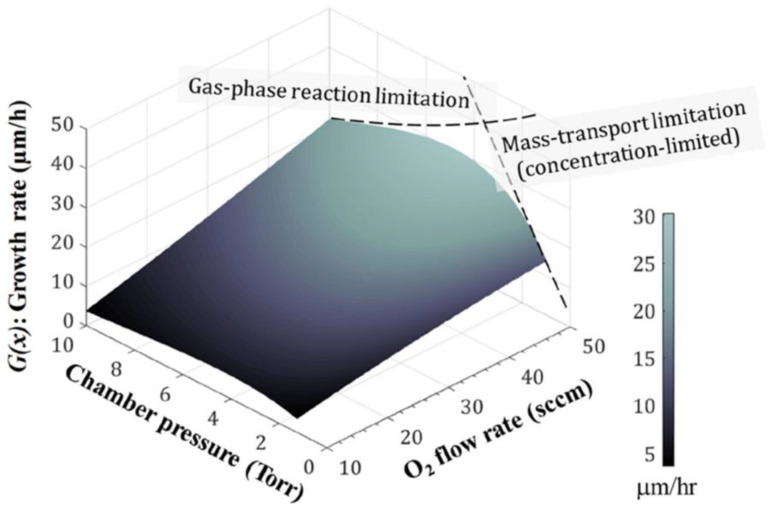
The growth rate of a Ga_2_O_3_ film was calculated using 3-D CFD with varying chamber pressures and oxygen-flow rates. The argon flow was kept at 300 sccm, and there was 5 cm between the substrate and the precursor. (Reprinted from ([244]).

The chamber pressure also highly affects the growth rate and the carrier concentration. Increased chamber pressure leads to a slower growth rate but a higher carrier concentration. This inverse relationship with the growth rate is related to limitations with the gas-phase reaction and the concentration. Figure 38 shows the simulation results comparing the effects of oxygen-flow rate and the chamber-pressure on the growth rate, assuming a fixed argon flow of 300 sccm, while the distance from the Ga precursor to the substrate is 5 cm.

#### 7.2.2. Growth on Other Substrates

LPCVD has also been used to grow Ga_2_O_3_ on substrates other than sapphire, such as 4H-SiC and diamond [233,234,235,248,249]. These substrates are popular for Ga_2_O_3_ growth due to their high thermal conductivity and their P-type doping. Ga_2_O_3_ lacks both these qualities, and this heterojunction would allow for better thermal management of the device, along with access to PN structures. For Ga_2_O_3_/4H-SiC heteroepitaxy, a FWHM of 1.37° and an RMS roughness of 2.25 nm have been reported [250]. It was found that an off-angle is helpful for Ga_2_O_3_ growth on 4H-SiC and for similar reasons as sapphire. Growing Ga_2_O_3_ on a diamond is challenging due to surface oxidation, but by employing a low-temperature Ga_2_O_3_ layer, an FWHM of 1.8°, an electron mobility of 3 cm^2^/V·s, and a growth rate of 5.1 µm/h have been achieved [235].

#### 7.2.3. Homoepitaxy

LPCVD can also grow Ga_2_O_3_ on its native substrate. On Ga_2_O_3_, the FWHM of the XRD rocking curve can reach 0.013°, the electron mobility can reach 156 cm^2^/V·s, the RMS roughness can reach below 3 nm, and the growth rate can reach over 20 µm/h [246,251,252].

There is a reduction in the surface roughness and growth rate at higher temperatures in many studies of homoepitaxy of Ga_2_O_3_, and it is thought to be caused by the creation of Ga_2_O and the subsequent expulsion due to the reaction at high temperatures of Ga with Ga_2_O_3_ [236,251]. However, similar to the LPCVD growth on sapphire, if the oxygen flow rate is increased, both the surface roughness and the growth rate increase as the temperature goes up [244]. The high-temperature growth was also shown to have less contamination of Cl than other growths done at lower temperatures, with the Cl concentration being below the detectible limit, proving that SiCl_4_ is a viable precursor for the dopant in an LPCVD growth system [244].

LPCVD can perform homoepitaxy on multiple planes without a significant decrease in growth rate compared to other methods, such as MOCVD and molecular beam epitaxy (MBE [102,251,252,253]. It was demonstrated that the (010) plane produces better crystal quality samples than the (001) plane, with the FWHM not changing from the original substrate. The growth rate and the electron mobility were both higher in the (010) plane compared to the (001) plane. However, the difference was small [254].

#### 7.2.4. Nanostructure Growth

**Figure 39:**
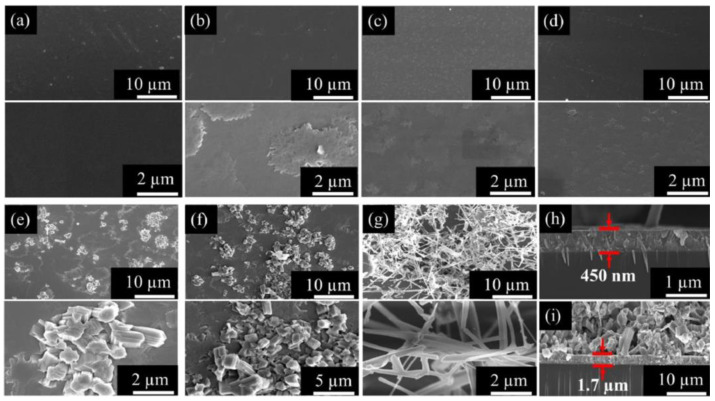
(**a**–**g**) The SEM images of the transition from a thin film to nanostructure based on Ga concentration in LPCVD of Ga_2_O_3_. (**h**,**i**) The cross-sectional SEM image of (**d**,**g**) (reprinted from [254]).

LPCVD is also capable of producing nanostructures, which can exploit quantum effects and nanoscale dimensions [254,255,256]. These special properties can be used for advanced applications such as deep UV photodetectors, nanophotonic switches, sensors, and field-effect transistors [257,258,259,260,261,262,263,264,265]. These devices are often grown using catalytic methods that may introduce contaminants [266,267,268,269,270]. However, LPCVD can produce Ga_2_O_3_ nanorods with lengths of up to 600 µm and diameters of approximately 30 nm [256]. Figure 39a–i shows the transition from single crystal film to nanostructure due to increased gallium concentration. Nano-electromechanical (NEM) devices have also been fabricated using LPCVD-grown Ga_2_O_3_ [271,272].

### 7.3. Summary and Future Prospects

LPCVD is a versatile and efficient method for the deposition of β-Ga_2_O_3_ thin films and nanostructures, offering high uniformity, low impurity levels, and scalability. Its ability to adapt to various substrates and growth conditions makes it a valuable tool in the fabrication of advanced electronic and optoelectronic devices. Further, the optimization of the growth process for power devices is needed to show the reliability of LPCVD. Achieving faster growth rates with high crystal quality is also a necessary area of research for LPCVD-grown Ga_2_O_3_ devices. Continued research is also needed in the high-temperature regime of LPCVD to see if the mobility, surface roughness, and growth rate will continue to improve as the temperature and the oxygen flow rate increase. The critical electric field of the HTLPCVD-grown Ga_2_O_3_ should also be tested and compared to LPCVD and other growing methods. Table 4 compares the different LPCVD growth cited in this paper.

**Table 4:** A comparison of the crystal quality based on the growth parameters.

Researcher	Temperature (°C)	Growth Rate (μm/h)	RMS Roughness (nm)	FWHM (°)	Hall Mobility (cm^2^/V·s)	O_2_ Flow Rate (sccm)	Pressure (torr)	Substrate to Crucible (cm)	Substrate
[93,230,243,251]	900	10	-	0.47	106.6	-	4	-	Sapphire
	900	1.9	1.3	0.013	76	-	4	-	Ga_2_O_3_ (010)
	900	1.2	4	0.043	42	-	4	-	Ga_2_O_3_ (001)
	800	1.7	-	-	42.35	-	-	-	sapphire
	950	1.3	16.9	-	-	-	-	-	Ga_2_O_3_
Joshi et al. [226,231]	825	3	6	-	-	20	1.5	12	sapphire
	875	6	4.56	2.1	-	5	0.75	5	sapphire
	925	6	5.35	1.5	-	5	0.75	5	sapphire
Feng et al. [244]	900	10–30	-	-	50–63	15–30	-	-	sapphire
	820–940	1.5–20	-	-	45–70	-	-	-	sapphire
	900	7–13.5	-	-	50–65	-	2–12	-	Sapphire
	900	14–10	-	-	-	15	1.7	2–7	sapphire
	1000	12	-	-	126	20	-	2	Sapphire
	1050	-	3	-	156	30	-	2	Ga_2_O_3_
Jiao et al. [273]	800–900	0.72	1.18–12	1.82–1.7	-	-	0.37	-	sapphire
Zhang et al. [246]	1000	12	-	-	126	20	-	2	Sapphire
	1050	-	3	-	156	30	-	2	Ga_2_O_3_
Wu et al. [247]	900	1.5	-	1.6	-	25	-	20	sapphire
Hu et al. [250]	650	-	2.25	1.37	-	-	0.75	-	4H-SiC
Akyoler et al. [245]	850–925	0.49–3.421	40–0.5	0.09–0.2	-	8	0.7	11.5	sapphire
Karim et al. [235]	920	5.1	-	1.1	5.1	15	-	-	Diamond
Ranga et al. [274]	800–900	22	-	-	20–5	3–5	0.5–2	-	Ga_2_O_3_

## 8. Discussion

MOCVD, MBE, and HVPE are still the predominant techniques used for the large-scale production of epitaxially grown films. MOCVD is a highly versatile and widely used technique for the epitaxial growth of β-Ga_2_O_3_ films, utilizing organometallic precursors such as Triethylgallium (TEGa) and Trimethylgallium (TMGa). This method is known for producing high-quality films with excellent structural and electrical properties. MOCVD allows for precise control over growth parameters, enabling the optimization of film thickness, growth rate, and doping concentrations. The close-coupled showerhead design in MOCVD systems enhances growth rates by minimizing gas-phase reactions between metal-organic sources and oxygen, achieving growth rates of up to 10 µm/h on sapphire substrates. However, managing gas-phase reactions remains a challenge, as higher growth rates can lead to increased defect densities and degraded material quality. Recent advancements include the development of high-quality β-(Al_x_Ga_1−x_)_2_O_3_ heterostructures and the successful incorporation of aluminum with rates up to 40% on native (010) substrates. MBE is renowned for producing β-Ga_2_O_3_ films with exceptional purity and defect-free epitaxial layers. It operates under ultra-high vacuum conditions, using pure metal and oxygen sources, which allows for precise control over doping profiles and growth parameters. MBE-grown films are highly regarded for their superior structural, optical, and electrical characteristics. Techniques such as plasma-assisted molecular beam epitaxy (PAMBE) and ozone-enhanced MBE (ozone MBE) have been employed to produce β-Ga_2_O_3_ with various doping and heterostructure schemes. However, MBE faces limitations in growth rates and film sizes, which can restrict its scalability for large-scale production. Recent innovations like indium-mediated metal-exchange catalyzed MBE (MEXCAT-MBE) and metal-oxide catalyzed epitaxy (MOCATAXY) have shown promise in enhancing growth rates and crystal quality. HVPE is characterized by its rapid growth rates and ability to produce thick β-Ga_2_O_3_ layers. This method utilizes inorganic precursors such as Gallium Chloride (GaCl) and operates at high temperatures. HVPE can achieve growth rates ranging from a few μm/h to approximately 250 μm/h, making it suitable for producing thick drift layers required for high-voltage devices. However, HVPE-grown films often exhibit significant surface roughness and contain chlorine-induced impurities. Efforts to optimize growth conditions focus on balancing high growth rates with film quality, and recent studies have demonstrated the growth of various Ga_2_O_3_ polymorphs, including α- and ε-phases, by tuning the growth temperature.

Table 5 presents data on Ga_2_O_3_ thin films grown on various substrates using different epitaxial techniques, detailing sample size, film thickness, inhomogeneity, standard deviation (SD) of thickness, and references. It includes samples of Ga_2_O_3_/SiO_2_/Si grown by PEALD on a 4-inch substrate with a thicknesses of 10–30 nm and inhomogeneity of 1.5–2% (Ref. [275]), Ga_2_O_3_/Si grown by MOCVD on an 8-inch substrate with a thickness of 90 nm and inhomogeneity of 3.3% (Ref. [276]), Ga_2_O_3_/Al_2_O_3_ grown by Mist CVD on a 4-inch substrate with a thickness of 230 nm and SD of 28 nm (Ref. [193]), Ga_2_O_3_/Al_2_O_3_ grown by Mist CVD on a 2-inch substrate with a thickness of 187 nm and inhomogeneity of 20% (Ref. [196]), Ga_2_O_3_/Al_2_O_3_ grown by Mist CVD on a 2-inch substrate with a thickness of 210 nm, inhomogeneity of 3%, and SD of 5.7 nm (Ref. [277]), Ga_2_O_3_/Al_2_O_3_ grown by HVPE on a 2-inch substrate with a thickness of 1100 nm, inhomogeneity of 2.6%, and SD of 50 nm (Ref. [278]), and Ga_2_O_3_/Al_2_O_3_ grown by MOCVD on a 2-inch substrate with a thickness of 1550 nm, inhomogeneity of 1.8%, and SD of 28 nm (Ref. [142]).

**Table 5:** Comparison of film parameters of Ga_2_O_3_ epitaxial growth [143].

Samples	Ga_2_O_3_/SiO_2_/Si	Ga_2_O_3_/Si	Ga_2_O_3_/Al_2_O_3_	Ga_2_O_3_/Al_2_O_3_	Ga_2_O_3_/Al_2_O_3_	Ga_2_O_3_/Al_2_O_3_	Ga_2_O_3_/Al_2_O_3_
Growth approach	PEALD	MOCVD	Mist CVD	Mist CVD	Mist CVD	HVPE	MOCVD
Size (in.)	4	8	4	2	2	2	2
Thickness (nm)	10–30	90	230	187	210	1100	1550
Inhomogeneity (%)	1.5–2	3.3	-	20	3	2.6	1.8
SD (nm)	-	-	28	-	5.7	50	28
Ref.	[275]	[276]	[193]	[195]	[277]	[278]	[142]

Growth Rate: HVPE offers the highest growth rates (up to 250 μm/h), followed by MOCVD (up to 10 μm/h) and MBE (up to 1 μm/h). Higher growth rates in HVPE are advantageous for producing thick layers but can compromise film quality.

Film Quality: MBE is superior in producing defect-free, high-purity films, making it ideal for applications requiring high precision. MOCVD strikes a balance between growth rate and film quality, making it versatile for various applications. HVPE, while fast, often results in films with higher roughness and impurities.

Scalability: MOCVD and HVPE are more scalable for industrial applications due to their higher growth rates and ability to produce large-area films. MBE, although precise, is less scalable due to its slower growth rates and smaller film sizes.

Doping and Heterostructures: MBE provides excellent control over doping profiles and the formation of heterostructures. MOCVD also offers good doping control and has shown significant advancements in producing complex heterostructures. HVPE is effective for growing thick layers with low background impurities but offers less precise doping control compared to MBE and MOCVD.

## Data Availability

The data presented in this study are available on request from the corresponding author.
